# Combined theoretical and experimental insights on DNA and BSA binding interactions of Cu(ii) and Ni(ii) complexes along with the DPPH method of antioxidant assay and cytotoxicity studies†

**DOI:** 10.1039/d2ra08341h

**Published:** 2023-03-08

**Authors:** Prasun Acharya, Arun Kuila, Ushasi Pramanik, Venkatesha R. Hathwar, Paula Brandao, Saptarshi Mukherjee, Swapan Maity, Tithi Maity, Ribhu Maity, Bidhan Chandra Samanta

**Affiliations:** a Department of Chemistry Mugberia Gangadhar Mahavidyalaya Bhupatinagar Purba Medinipur-721425 West Bengal India bsmgm1977@gmail.com bidhansamanta@yahoo.in +91-3220-270236; b Department of Chemistry, IISER Bhopal Bhopal Bypass Road, Bhauri Bhopal 462 066 Madhya Pradesh India; c School of Physical and Applied Sciences, Goa University Taleigao Plateau Goa 403 206 India; d Departamento de Química, CICECO, Universidade de Aveiro 3810-193 Aveiro Portugal; e School of Materials Science and Technology (SMST), Indian Institute of Technology (IIT), BHU India; f Department of Chemistry, Prabhat Kumar College Purba Medinipur-721401 Contai West Bengal India

## Abstract

This present study delineates the syntheses, detailed characterization and anti-proliferative potential against SiHa (cervical cancer cell) of two mononuclear complexes of Cu(ii) and Ni(ii) using a Schiff base ligand (L) derived from 2-hydroxybenzaldehyde and *N*-methyl-propane 1,3-diamine. The crystallographic results show the centro-symmetric space group of orthorhombic nature (*Pccn*) for Cu(ii) complex (1) where the central Cu(ii) has an inversion center symmetry with six co-ordinations resulting in a distorted octahedral geometry. Whereas, in complex (2), the two independent Ni(ii) atoms present in the special position within version symmetry and form a distorted geometry of octahedral nature with six coordinations. Absorption spectral titrations with Calf Thymus (CT) DNA and the extent of the decrease in relative emission intensities of DNA-bound ethidium bromide (EB) upon adding the complexes reveal the parallel trend in DNA binding affinities for both the complexes but with a small extent of binding capabilities. Bovine serum albumin (BSA) interaction studies demonstrate that complex 1 exhibits more promiscuous binding with BSA as compared to complex 2 from the spectroscopic and theoretical approaches. α,α-Diphenyl-β-picrylhydrazyl (DPPH) free radical scavenging method shows a little antioxidant or free radical scavenging activity for both the studied complexes. Cytotoxicity studies against SiHa expressed that the percentage of cell viability was reduced with time whereas in the same concentration and conditions, the viability percentage was higher for 3T3-L1 (several normal cell lines of mouse). The fluorescence imaging obtained from acridine orange (AO) and ethidium bromide (EtBr) demonstrates that the colour of the cancer cells has changed gradually dictating the cell apoptosis from day 1 to day 3.

## Abstract 2

Combined theoretical and experimental insights on DNA and BSA binding interactions of Cu(ii) and Ni(ii) complexes along with DPPH method of antioxidant assay and cytotoxicity studies.

## Graphical abstract

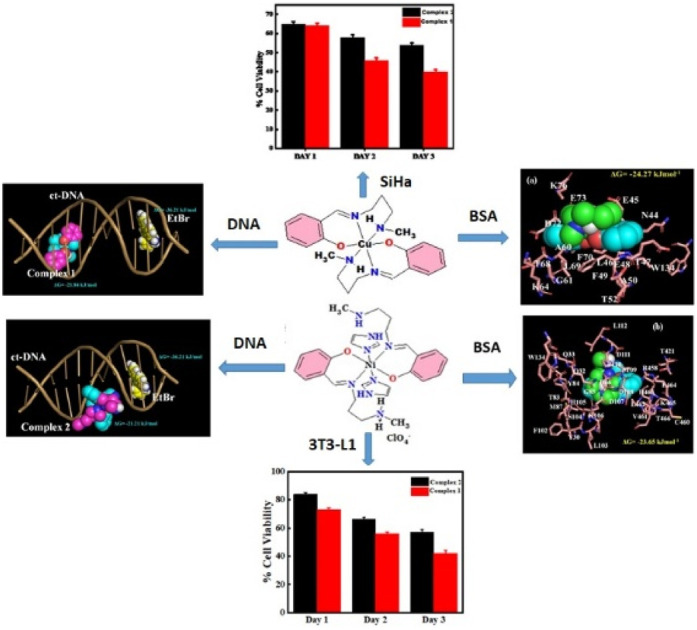

## Introduction

Co-ordination complexes of transition metals acquire notable anti-tumor, anti-microbial properties and provide broad opportunities in the field of drug discovery controlled by varieties of ligand, metal or changes in oxidation states of metal ions during coordination.^1^ The foremost privilege of these metal based drugs over organic pharmaceuticals is the capability of those compounds to adjust coordination number, redox states and geometry.^2^ Hence researchers are interested to use the transition metals as core constituent elements in the group of developed medicines, which in turn increase the significance of synthesizing metal complexes. Additionally, Schiff base complexes are of special interest in the bioinorganic field due to their chelation property^3,4^ and tuning efficacy between anti-tumor activity and substitutions used in the aromatic moieties.

Over the past decades, cisplatin, the platinum-based drug has been extensively used efficiently in antitumor remedy but high toxicity and side effects have limited its use to patients. Copper^5–8^ and ruthenium^9–15^ complexes show potential anticancer properties and are considered as alternatives to platinum complexes. Copper, among the transition metals, is significantly essential in +2 oxidation states because in this state it has a vital responsibility in DNA damage linked to cancer^16^ and demonstrates general toxicity lower than platinum. So some Cu(ii) co-ordination complexes are now projected as prospective tumor inhibiting substances.^17–19^ Not only Cu, nickel (Ni) complexes are also still emerging in this respect because of its exciting structural aspects, low cost, low toxicity and easy availability.^20,21^ Herein, we have reported the syntheses of Cu(ii) and Ni(ii) complexes containing Schiff base ligand of 2-hydroxybenzaldehyde and *N*-methyl-propane 1,3-diamine along with their characterizations through several experimental and theoretical approaches.

Researchers have also developed profound interests to decipher the interaction studies of those metal complexes with biomolecules *viz.* DNA^22–25^ and proteins.^26–28^ These investigations provide detailed information regarding the drug transporting, metabolism and promote the development of new metallo-pharmaceuticals. Consequently, the interaction study of metal-based complexes with albumin and DNA is one of the best models to get primary insights regarding the binding interactions of those complexes with biomolecules. DNA being the key target molecule for the majority of anticancer and antiviral therapies, thus, to develop prospective DNA targeting anti-proliferative drugs, the binding interactions of metal complexes to DNA are planned. In this regard, bovine serum albumin (BSA) has also been extensively used during the last decades due to its structural homology with HSA (Human Serum Albumin).^29,30^ As antioxidants effectively combat the free radicals which are known to cause various degenerative disorders, like mutagenesis, carcinogenesis, cardiovascular disturbances and ageing, so, it is important to measure antioxidant capacity that is free radical scavenging activity of a compound to be used as antitumor drug.^31,32^ α,α-Diphenyl-β-picrylhydrazyl (DPPH) free radical scavenging method offers the first approach for evaluating the antioxidant potential of a compound. It is a rapid, simple, inexpensive and widely used method to measure the ability of compounds to act as free radical scavengers.^33^

In this context, we have reported DPPH method for determining antioxidant capacity of the studied complexes along with binding interactions of those complexes with DNA and BSA using various spectroscopic techniques. The *in vitro* cytotoxic studies against SiHa cancer cells and 3T3-L1 normal cells have also been investigated in the present study.

## Experimental section

### Materials

Materials used for the experiments done in the current work were employed in the experiments without further purifications. From Merck, 2-hydroxybenzaldehyde, *N*-methyl propane 1,3-diamine, imidazole, methanol, CuCl_2_·2H_2_O, Ni(ClO_4_)_2_·6H_2_O *etc.* were procured. BSA and DNA was purchased from Sigma-Aldrich Chemicals (USA) and was prepared in 10 mM Phosphate Buffer (pH 7.4). For steady-state and time-resolved experiments, the concentration of BSA was kept as 5 μM, whereas 2 μM BSA was used for Circular Dichroism (CD) spectroscopic studies.

### Ligand (L) synthesis

**Scheme 1:**
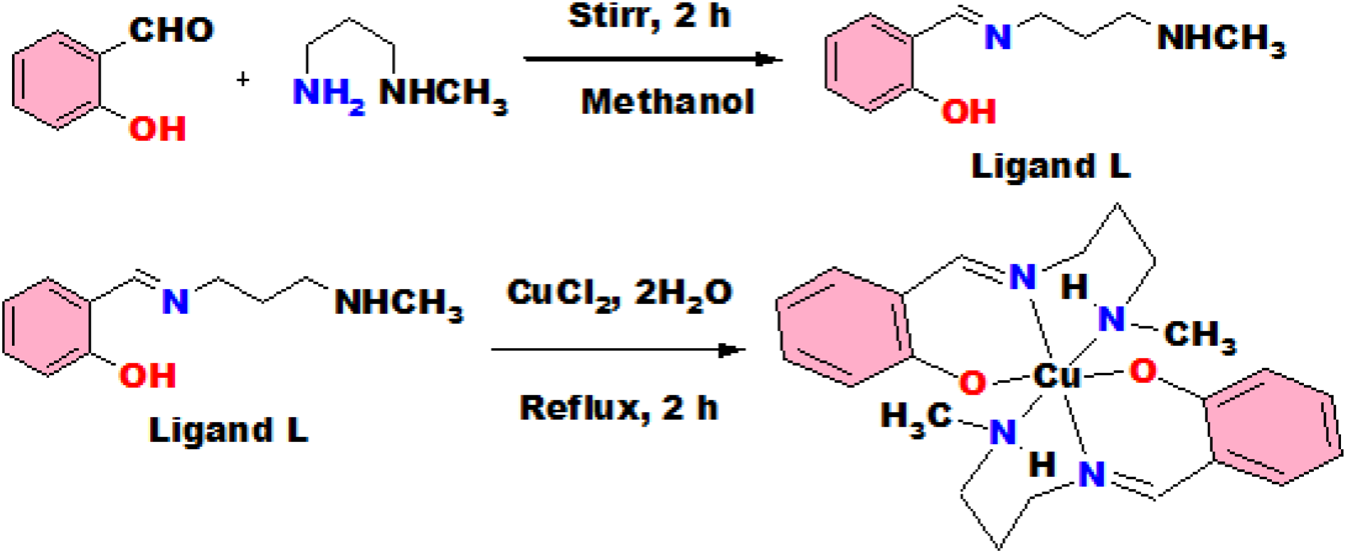
Reaction details for preparation of ligand and complex (1).

It was synthesized by stirring the mixture of 2-hydroxybenzaldehyde and *N*-methyl propane 1,3-diamine taking 1 mmol each in methanol for 2 h. The reaction mixture was transformed into a yellow solution and employed directly for the preparation of complexes (Scheme 1).

### Synthesis of complex (1)

Complex 1 was prepared by a refluxing mixture of CuCl_2_·2H_2_O (0.5 mmol) and L (1 mmol) for about 3 h in methanol. A green colour solution was observed and allowed to evaporate slowly (Scheme 1). Few days later, single crystals of green colour were produced from slow evaporation technique and employed for X-ray diffraction study.

Yield: 85%. Molecular formula C_22_H_30_CuN_4_O_2_; characteristic FTIR peaks (KBr, cm^−1^; br = broad, s = strong, m = medium, vs = very strong): 1639 (m), 1317 (s), 1209 (s). UV absorption in MeOH [*λ*_max_, nm]: 375. Mass spectra *m*/*z*: expected for [C_22_H_30_CuN_4_O_2_]^+^ 445.55, observed 445.18.

### Synthesis of complex (2)

**Scheme 2:**
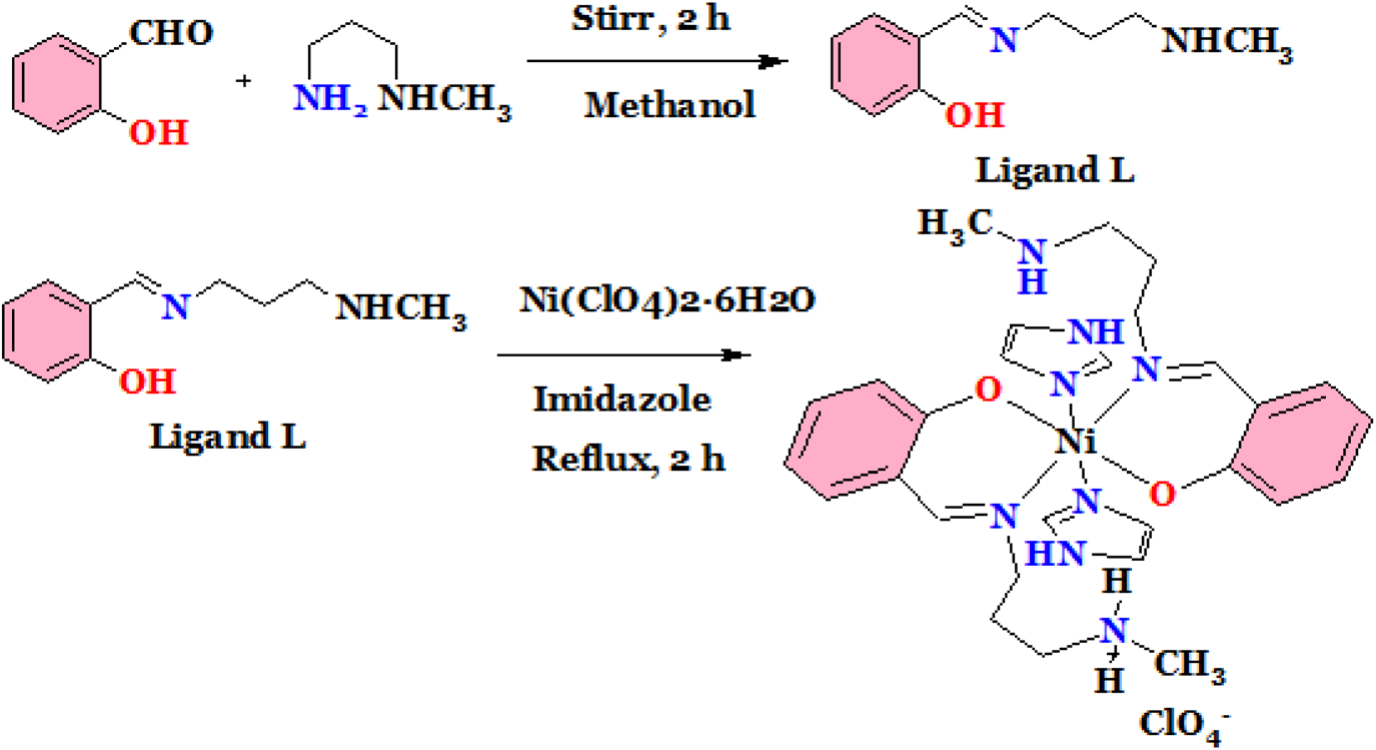
Reaction details for preparation of complex (2).

This was synthesized by reacting the mixture of Ni(ClO_4_)_2_·6H_2_O (0.5 mmol), ligand (1 mmol) and imidazole (1 mmol) under reflux condition for about 3 h in methanol. A light brown colour solution was observed that was allowed to evaporate slowly. Few days later, single crystals were produced from slow evaporation technique and employed for X-ray diffraction study (Scheme 2).

Yield: 86%. Molecular formula C_28_H_39_N_8_NiClO_6_; characteristic FTIR peaks (KBr, cm^−1^; br = broad, s = strong, m = medium, vs = very strong): 1648 (s), 3235 (m), 1442 (s), 1317 (s), 1203 (s), 3158 (m), 2950 (m). UV absorption in MeOH [*λ*_max_, nm]: 372. Mass spectra *m*/*z*: expected for [C_28_H_39_N_8_NiClO_6_]^+^ 677.19, found 677.49.

### Characterizations

FTIR study was performed using KBr plate in Shimadzu IR Affinity – 1S spectrometer. For UV studies, UV-Vis spectrophotometer (Systronic, India) was employed. Mass spectroscopic study of the complex was carried out in Waters XEVO G2-XS QTOF mass spectrometer.

### Crystallographic studies by X-ray diffraction

Crystal data collection was performed at 150 K by a graphite monochromated X-ray diffractometer (Bruker Kappa) with Mo Kα radiation of *λ* = 0.71073 Å. APEX-II (v2.0-2) program (Bruker) was employed for data processing using φ and ω-scan techniques. For data correction towards Lorentz and polarization effects, SADABS program^34^ was used. SHELXT 2014/5 was utilized for solving the structure by direct method and subsequent refinement was performed with the help of *F*^2^ by means of SHELXL2018/3.^35^ Refinement of non-hydrogen atoms was done including parameters related to anisotropic displacement. Thermal parameters used for refinement of hydrogen atoms are 1.2 or 1.5 times greater than subsequent mother atoms. Table 1 shows data and parameters related to crystallographic studies of the complexes. The CCDC number, 2179951 (complex 1) and 2179957 (complex 2) are gained from CCDC Centre (Cambridge).

**Table 1:** Crystallographic and structural details of the complexes

Crystal data	Complex (1)	Complex (2)
Chemical formula	C_22_H_36_CuN_4_O_5_	C_50_H_70_Cl_2_N_12_Ni_2_O_12_
Molecular weight	500.09	1219.50
Crystal system	Orthorhombic	Monoclinic
Space group	*Pccn*	*P*2_1_/*c*
Wavelength (Å)	0.71073	0.71073
*a* (Å)	16.0184(6)	10.5595(5)
*b* (Å)	17.2661(7)	12.9431(7)
*c* (Å)	8.8793(4)	20.8682(11)
*α* (°)	90	90
*β* (°)	90	98.743(2)
*γ* (°)	90	90
*V* (Å^3^)	2455.79(17)	2819.0(3)
*Z*	4	2
*T* (K)	150	150
*μ* (mm^−1^)	0.93	0.83
Crystal size (mm)	0.18 × 0.08 × 0.04	0.26 × 0.18 × 0.04
*T*_min_, *T*_max_	0.851, 0.928	0.813, 0.967
Measured reflections	68 421	79 737
Unique reflections	2719	6226
Observed reflections [*I* > 2*σ*(*I*)]	2127	5407
*R*_int_	0.049	0.032
*θ*range	2.36–27.15	1.86–27.13
*R*[*F*^2^> 2*σ*(*F*^2^)], w*R*(*F*^2^), *S*	0.040, 0.122, 1.129	0.035, 0.090, 1.071
No. of parameters	164	373
H-atom treatment	Constrained	Constrained
Δ*ρ*_max_, Δ*ρ*_min_ (e Å^−3^)	0.45, −0.72	0.92, −0.96

### Hirshfeld surface (HS) analyses

The quantitative analysis of intermolecular interactions in the crystal structure is obtained by HS analysis using Crystal Explorer 3.1 software.^36^ The HS analysis involves the partition of electron density (ED) of a molecule into atomic fragments such that a molecule within a crystal is given by a weighting function
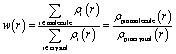
where *ρ*(*r*) is a spherically averaged Hartree–Fock atomic ED function of *i*^th^ nucleus. The cutoff of the weight function is 0.5 Å.

Further, the visualization of different interactions in the crystal structure is performed using different functions such as *d*_e_, *d*_i_, *d*_norm_, shape index and curvedness mapped on the HS of the molecules. The normalized contact distance (*d*_norm_) in the HS analysis is given by
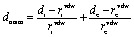
*d*_e_ denotes HS distance from nearest nucleus situated outside surface, *d*_i_ is the HS distance from nearest nucleus situated inside surface and *r*^vdw^ corresponds to van der Waals radius. The *d*_norm_ parameter on the HS of the molecule is visualized in a red-white-blue color code. The corresponding intermolecular contacts which are less than their vdW radii are indicated by red regions on the HS. Whereas the blue regions show intermolecular contacts of distance longer than their vdW radii. White regions signify that the contact distance is identical to the summation of vdW radii. Complementary nature of intermolecular interactions during the packing of molecules is identified by shape index. The curvedness is also useful parameter to understand the interactions such as π⋯π interactions. The flat regions of a surface are highlighted by a low value of curvedness. Similarly, the 2D fingerprint plot associated with the HS give quantitative information on the percentage contribution of individual interactions to the supramolecular assembly.

### Interaction studies with BSA

#### Steady-state experiments

The steady-state measurements were performed by UV-vis spectrophotometer (Cary 100) and Fluorolog 3-111 (Horiba JobinYvon). Complex 1 has absorption centered around 375 nm (Fig. S8c†), while complex 2 shows absorption around 372 nm (Fig. S8d†). The fluorescence spectral intensities were modified to avoid the inner filter effect coming from significant absorption of complex below 300 nm (along with the absorption maximum at 370 nm) by using eqn (1):^37^

1 *F* = *F*_obs_ × antilog^(*A*_ex_+*A*_em_)/2^

#### Lifetime studies

Time resolved studies were recorded in time-correlated single-photon counting (TCSPC), *λ*_ex_ = 295 nm (fwhm ∼ 800 ps). With DAS-6 software obtained data were deconvoluted by using eqn (2):^37^2
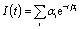
*α*_*i*_ represents the amplitude of the *i*^th^ lifetime *τ*_*i*_. The average lifetime 〈*τ*〉 was anticipated by eqn (3):3
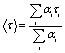


#### CD spectroscopic study

The CD spectra were taken in a spectropolarimeter (JASCO J-815) after correcting baseline at 298 K and using a 0.1 cm cuvette of quartz. The spectra were recorded within the wavelength range of 200–260 nm with a scanning rate of 100 nm min^−1^, and each spectrum was as an average of three scans. The obtained ellipticity (*θ*) was expressed in MRE (Molar Residual Elipticity):^38^4
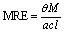
Here, *M* and *a* denote molecular weight of protein and the number of amino acid residues, respectively. *c* is the concentration in g L^−1^, and *l* is the path length.

#### Binding interaction studies with DNA by UV absorption and fluorescence techniques

UV absorption titration was employed to study the binding interactions of the complexes with DNA in a UV-vis spectrophotometer (Cary 100) using reference Phosphate Buffer. The emission spectra were recorded in Fluorolog 3-111 (Horiba JobinYvon) spectrophotometer by noting the changes of fluorescence intensities of the ethidium bromide-DNA adduct with gradual addition of the complexes.

#### Docking studies

AutoDock 4.2 software was utilized for performing the semi-rigid docking studies.^39^ The highest binding energy is well thought-out as the most excellent docked conformer of the ligand. The BSA structure was taken from RCSB protein data bank (PDB ID 3V03) and coordinates of CT-DNA was also downloaded from RCSB website. The ligand molecule (Complex) was optimized in Gaussian 09 software and the optimized structure was further using in docking.^40^ The grid box sizes along three axes were kept as 126 while the grid spacing was 0.375 Å. The conformation of most excellent docked conformer was examined using PyMOL software^41^ out of 50 different conformations. The grid center coordinates were 64, 25 and 32 along *X*-, *Y*- and *Z*-directions. The population size was kept as 150 in Genetic Algorithm (GA) and the maximum number of energy evaluations was set as 250 000.

#### *In vitro* antioxidant activities studies by DPPH method

DPPH free radical scavenging assay method was employed to study the potential antioxidant activities of the complexes (1 and 2) using a known procedure.^42^ 51 μM DPPH solution in methanol was prepared. Different concentrations of each complex solution in DMSO were added to 5 mL of DPPH solution. The mixture was shaken vigorously and incubated in dark room temp for 30 min. Decrease in absorbance of DPPH centered at 517 nm was then measured for both the complexes along with ascorbic acid (AA) as standard (Fig. S13a, S14a & S15a)† and the colour changes of pure DPPH solution with gradual addition of AA and complexes were noted (Fig. S13b, S14b & S15b).† The scavenging activity % of DPPH was calculated by using following formula:Scavenging activity (%) = [(*A*_0_ − *A*_1_)/*A*_0_] × 100where *A*_0_ is the absorbance of DPPH in absence of an antioxidant and *A*_1_ is the absorbance of DPPH in the presence of an oxidant. The 50% activity (IC50) was calculated using the percentage of activity.

### Cytotoxicity studies

#### Culture of cell and maintenance

Dulbecco's Modified Eagle Medium (DMEM) along with 10% heat-inactivated fetal bovine serum (FBS), 100 U mL^−1^ penicillin, and 100 μg mL^−1^ streptomycin were involved to culture SiHa cells (cancerous tissues of the cervix uteri) and 3T3-L1 (several normal cell lines of mouse). 310 K temperature was maintained during culture of the cells in a CO_2_ incubator with a 5% CO_2_ supply.

#### Cell proliferation assay

With the help of MTT assay (3-(4,5-dimethylthiazol-2-yl)-2,5-diphenyltetrazolium bromide), the cell proliferation was investigated. Seeding of cell was performed in 0.1 mL of DMEM consisting of 10% FBS, 50 U mL^−1^ penicillin and 50 μg mL^−1^ streptomycin at the confluences of 70–80% cells per well. These were incubated at 310 K in 5% CO_2_. For accuracy in the obtained results all the treatments were performed in triplicate. To remove the dead cells, media of each well were substituted by 100 μL fresh media. Then in order to produce water-insoluble formazan, 0.5 mg mL^−1^ of MTT solution in DMEM was supplemented to every well and incubated for extra 3 h at 310 K. This formazan of each well is then mixed with DMSO and absorbance was recorded at 570 nm by a micro plate reader. By using the following formula, percentage of cell viability was calculated.5
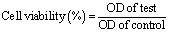
where, OD represents specimen optical density.

#### Fluorescence imaging

A fluorescence microscope was used to visualize cell proliferation efficiency on the specimen. Both cancer and normal cells were seeded onto the sample in 12-well plates for 24 h at 310 K. Fresh solution of PBS (phosphate-buffer saline) was used twice to wash test samples for removing the dead or floating cells. The 4% paraformaldehyde solution fixed the adhered cells for 20 min. Then they were washed again with PBS and marked by AO (acridine orange) and EtBr (ethidium bromide) (100 μg mL^−1^) fluorescent dye for 10 min. Again, these were rinsed with PBS for twice and consequently incubated for 5 min in dark. After that with a fluorescence microscope (Leica, Germany) images were collected.

## Results and discussions

### Characterization of the complexes

The studied complexes were prepared according to the procedures as described in the Schemes 1 and 2 and spectroscopic characterizations were performed using FTIR and ESI-MS spectrophotometers. From X-ray single crystal studies, crystal structures were identified.

The FTIR spectrum is shown in Fig. S1† for the complex from which it is observed that the strong peak for azomethine (C=N) group obtained at 1639 cm^−1^ indicating the formation of Schiff base. The band at 1317 cm^−1^ is assigned for aromatic C=C stretching and peak at 1209 cm^−1^ is due to C–C stretching. The Fig. S2† shows the FTIR spectrum for complex 2 from which it is observed that azomethine (C=N) group appears at 1648 cm^−1^. The bands at 3235 cm^−1^ and 1442 cm^−1^ are assigned for stretching and bending frequencies of N–H respectively. The characteristic stretching peaks for aromatic C=C, C–C, aromatic C–H and aliphatic C–H are observed at 1317 cm^−1^, 1203 cm^−1^, 3158 cm^−1^ and 2950 cm^−1^ respectively which strongly support the formation of the said complex. The *λ*_max_ values of 375 and 372 nm for complex 1 and 2 in the UV spectra are due to the π–π* transitions of aromatic rings and azomethine groups.

The ESI-MS spectrum of the complexes 1 and 2 are shown in Fig. S3 and S4† respectively. From the spectra, it is noted that prominent peaks for the possible *m*/*z* fragmented values of the complexes are observed which support the crystallographic structures of the complexes.

### Structural analyses for complex 1

**Fig. 1:**
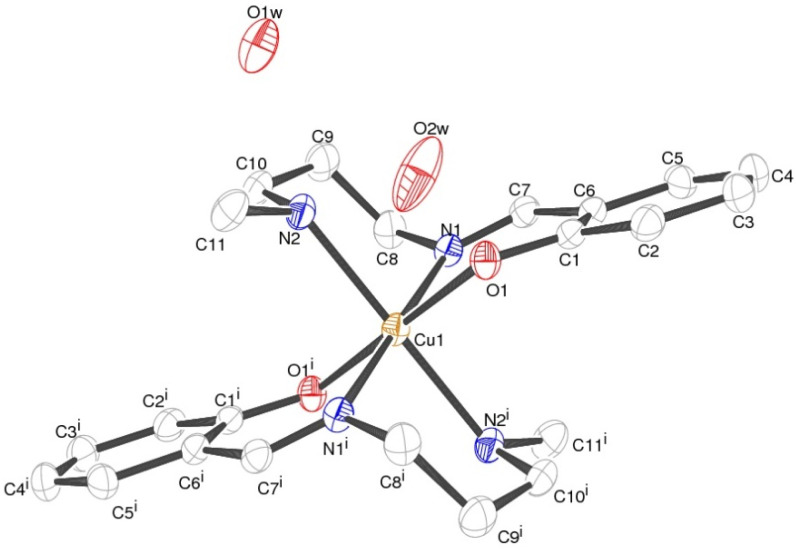
Crystal structure of complex 1 (ORTEP view) with 50% displacement ellipsoids.

**Fig. 2:**
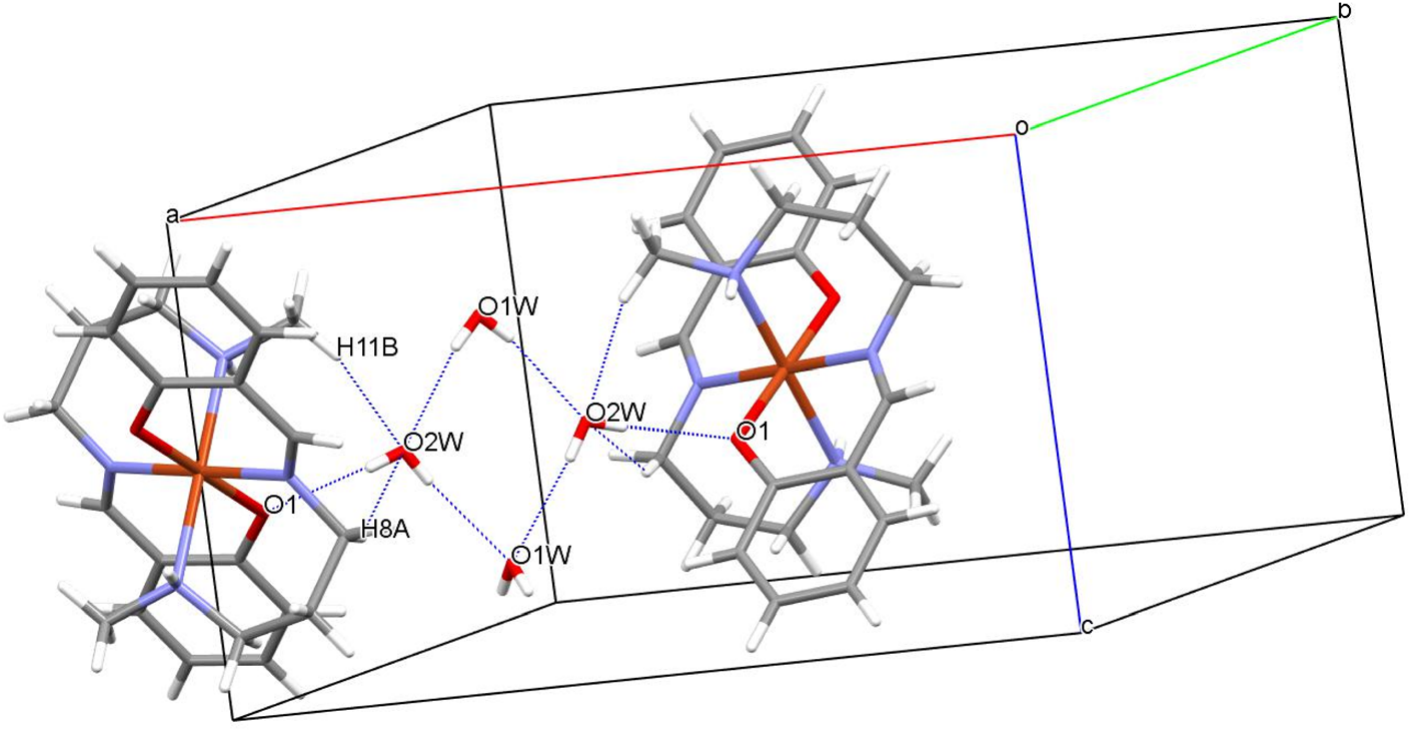
Representation of various interactions present in unit cell of complex 1.

Crystallographic studies reveal the centro-symmetric space group of orthorhombic nature (*Pccn*) for the complex (1) in which a central Cu(ii) is occupying an inversion center symmetry with six co-ordinations resulting in a distorted geometry of octahedral nature (Fig. 1). Table 1 shows all details regarding crystallographic and structural refinement. In the crystal, two water molecules are present and one of them is found on the 2-fold rotational axis. The Cu1–N2 is longer [2.184(2) Å] than the Cu1–N1 and Cu1–O1 bond lengths [2.050(2) Å] (Table S1†). The N–Cu–N, and O–Cu–N bond angle bond angles are in the range between 82.78(8)–87.13(8)° indicating that the {CuN_4_O_2_} octahedron is distorted (Table S1†). Hydrogen bonded water molecules form a channel along the ‘*c*’ axis. Fig. 2 depicts the H-bonding and the data are listed in Table 2. Hydrogen bonds from water molecules are strong and H⋯O distances vary from 2.075(3) to 2.176(5) Å. Further, water molecules are held by the molecular framework by feeble C–H⋯O interactions as shown in Fig. 2. The percentage contribution for various interactions obtained from HS fingerprint plot is shown in Fig. S5a.† Further, Fig. S6† expresses mapping of *d*_norm_, shape-index and curvedness properties.

**Table 2:** Details of interactions shown in complex 1

Interactions	Interaction distance *d* (Å)	Angle *θ* (°)	Symmetry
O2W–H2WA⋯O1	2.089(5)	174.4(1)	*x*,*y*,*z*
C11–H11B⋯O2W	2.637(3)	143.6(1)	*x*,*y*,*z*
O2W–H2WB⋯O1W	2.176(5)	175.1(1)	*x*,−*y* + 1/2,+*z* + 1/2
O2W–H2WB⋯O1W	2.176(5)	175.1(1)	−*x* + 1/2 + 1,+*y*,+*z* + 1/2
O1W–H1W⋯O2W	2.075(3)	168.2(1)	−*x* + 1/2 + 1,−*y* + 1/2,+*z*
C8–H8A⋯O2W	2.598(1)	127.9(1)	−*x* + 1,−*y* + 1,−*z* + 1

### Structural analyses for complex 2

**Fig. 3:**
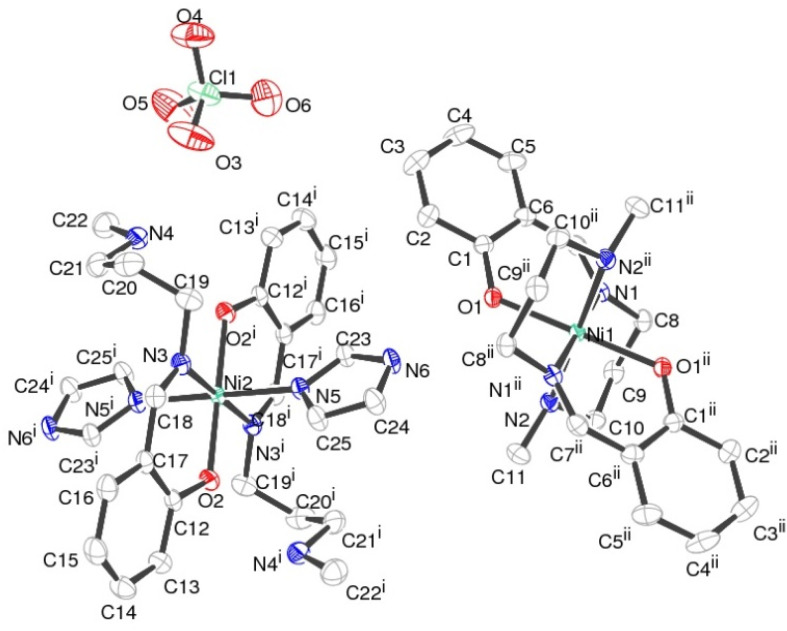
ORTEP plot of complex 2 with 50% displacement probability.

**Fig. 4:**
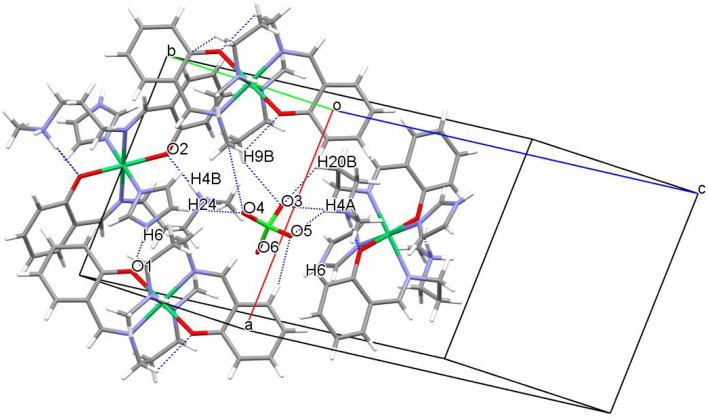
Intermolecular interactions in complex 2 unit cell.

The crystallographic studies show that complex 2 possesses *P*2_1_/*c* space group which is centrosymmetric and monoclinic in nature. There are two independent Ni(ii) atoms present in the special position within version symmetry and resulting in a distorted octahedral geometry with six coordinations in the crystal structure (Fig. 3). Further, the charge is balanced by a perchlorate ion in the crystal structure. One of the Ni–N bond is longer [2.181(2) Å] than the remaining Ni–N and Ni–O bond lengths [about 2.046(2)Å] (Table S2†). The N–Ni–N, and O–Ni–N bond angle bond angles are in the range between 83.23(6)–89.05(6)° indicating that the {NiN_4_O_2_} octahedron is distorted (Table S2†). The perchlorate ion forms many structure stabilizing hydrogen bonds. Among them, hydrogen bonds consisting of N–H⋯O are strong with N–H⋯O angles of 175.0(1)–178.0(1)° and H⋯O distances of 1.731(3)–1.860(3)Å. Further, weak C–H⋯O interaction (Fig. 4 and Table 3) stabilizes the crystal structure. The percentage contribution for various interactions obtained from HS fingerprint plot is shown in Fig. S5b.† The H⋯O interactions constitute about 23.3% of total interactions present the packing of molecules. Fig. S7† expresses mapping of *d*_norm_, shape-index and curvedness properties.

**Table 3:** Interaction details in complex 2

Interactions	Interaction distance *d* (Å)	Angle *θ* (°)	Symmetry
N4–H4B⋯O2	1.731(3)	178.0(1)	−*x* + 1,−*y* + 1,−*z* + 1
N6–H6⋯O1	1.860(3)	175.0(1)	−*x* + 1,−*y* + 1,−*z* + 1
C8–H8A⋯N6	2.817(2)	146.1(1)	−*x* + 2,−*y* + 2,−*z* + 1
C5–H5⋯O5	2.536(3)	162.0(2)	−*x* + 2,+*y* + 1/2,−*z* + 1/2
C9–H9B⋯O3	2.625(2)	145.8(1)	*x* + 1,−*y* + 1/2 + 1,+*z* + 1/2
C24–H24⋯O4	2.484(2)	145.9(1)	*x*,−*y* + 1/2 + 1,+*z* + 1/2
C19–H19A⋯O2	2.407(1)	124.1(1)	−*x* + 1,−*y* + 1,−*z* + 1
C13–H13⋯O6	2.770(3)	146.3(1)	−*x* + 1,−*y* + 1,−*z* + 1

### Interaction studies of the complexes with BSA

**Fig. 5:**
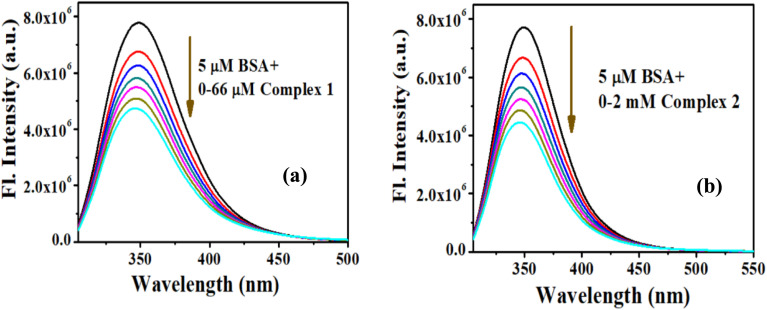
Emission spectra of BSA (5 μM) with rising concentrations of (a) complex 1 and (b) complex 2.

BSA has strong absorption at ∼280 nm which shows increment in the presence of complex 1 (Fig. S8a†) and complex 2 (Fig. S8b†). Upon excitation at 295 nm Tryptophan (W) of BSA elicits fluorescence emission centered at 350 nm,^43–47^ which shows reduction in its intensity (Fig. 5a and b) with successive addition of both the complexes as a result of quenching.^37^

**Fig. 6:**
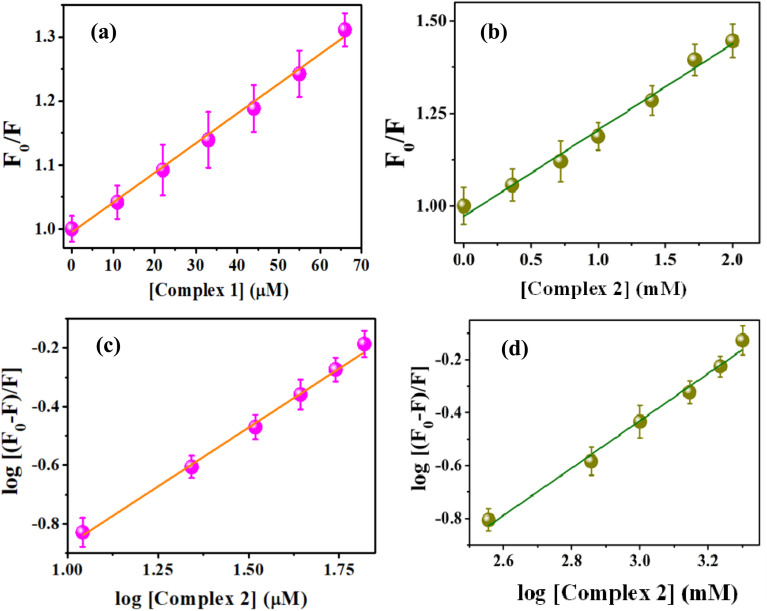
SV plot of interaction between BSA with (a) complex 1 and (b) complex 2. Double log plot of interaction between BSA with (c) complex 1 and (d) complex 2.

Quenching of fluorescence intensity can be attributed to the ground-state complex formation between the quencher and fluorophore or excited-state collision/diffusion between fluorophore and quencher, which can be analyzed by using the Stern–Volmer equation:^37^6
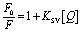
*F*_0_ and *F* represent the fluorescence intensities of the fluorophore (Tryptophan here) in the absence and presence of quencher *Q* (here the complexes). The plots (Fig. 6a and b) showing *F*_0_/*F* as a function of quencher concentration are linear nature, from which the Stern–Volmer constant for quenching has been determined (Table S3†).^37^ The linearity of the plots predicts one type of quenching phenomenon in this present study. The associated binding parameters between BSA and the complexes were obtained (Fig. 6c and d) from the equation below and the parameters were tabulated in Table S3:†7
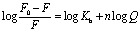
*K*_b_ represents the binding constant and *n* denotes the number of binding sites. The obtained binding parameters (Table S3†) resemble that the binding constant (*K*_b_) is greater for complex 1 with BSA than complex 2. These steady-state results delineate that the binding efficacy of complex 1 is more than complex 2 with BSA.

**Fig. 7:**
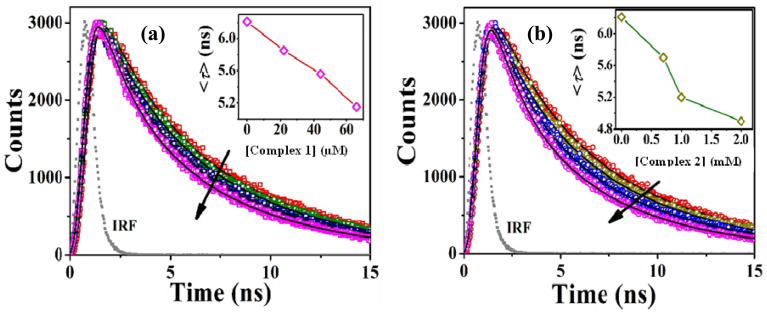
(a) Lifetime decay profiles of 5 μM BSA with rising concentrations of complex 1: 0 μM (red), 22 μM (green), 44 μM (blue) and 66 μM (pink) complex 1 as marked in the figure. Inset represents the change in average lifetimes of tryptophan in BSA in the absence and presence of different concentrations of the complex 1. (b) Lifetime decay profiles of 5 μM BSA with increasing concentrations of complex 2 as marked in the figure. [0 μM (red), 0.7 μM dark yellow, 1 mM blue and 2 mM pink].

To elucidate the exact nature of binding phenomenon, we further performed time-resolved studies. The excited-state lifetime of tryptophan in BSA decreases with rising concentrations of complex 1 and 2. The reduction in the lifetime dictates the quenching nature is a result of the excited-state collision of BSA with the complexes (Fig. 7a and b, Tables S4 and S5†). A plot of *F*_0_/*F* or *τ*_0_/*τ* as a function of the concentration of complexes (Fig. S9a and b†) follows similar trend. This observation also depicts that the quenching of BSA occurs through excited-state collision or diffusion with the said complexes. Thus, it can be concluded that the quenching governing the said interaction is dynamic in nature.^37^

**Fig. 8:**
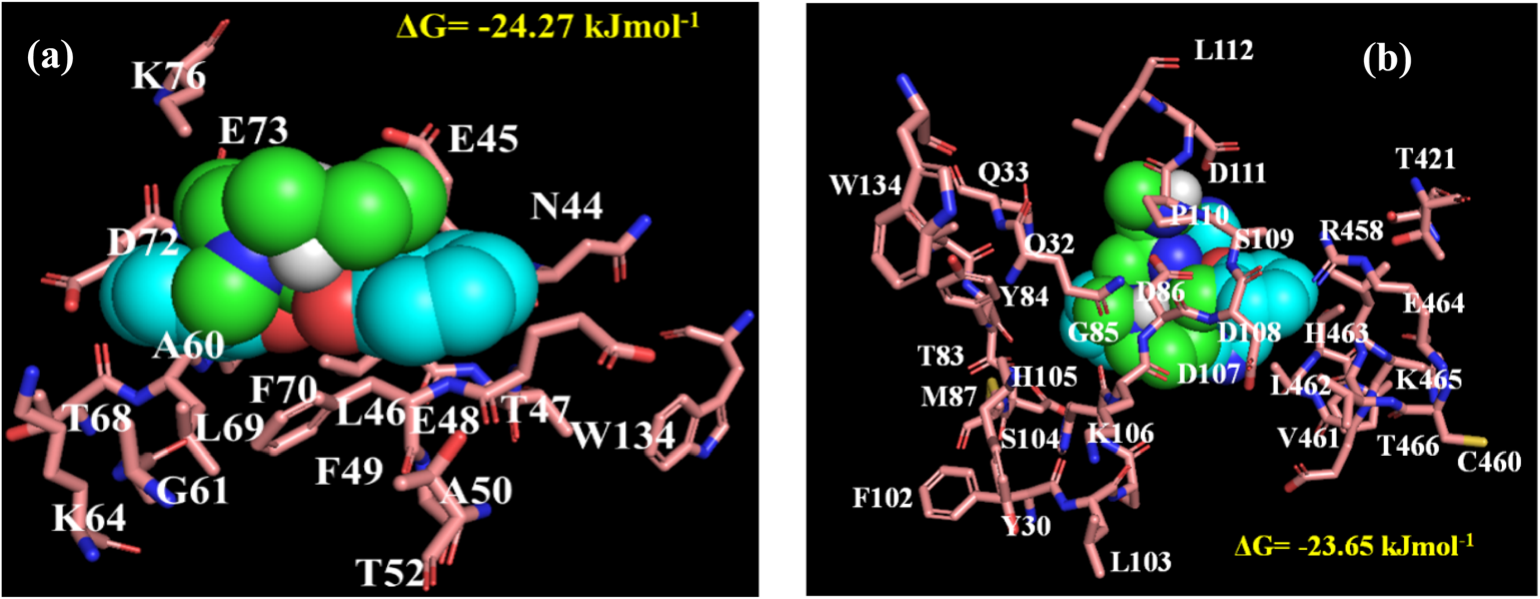
The amino acid residues of BSA within 8 Å distance from (a) complex 1 and (b) complex 2.

Furthermore, we have investigated the changes in the conformation of the protein upon the said interactions by circular dichroism (CD) spectroscopy. BSA shows helical nature in the CD spectra (Fig. S10a and b†) with two negative ellipticity bands at 222 nm and 208 nm corresponding to n → π* and π → π* transitions, respectively.^44^ It is important to note that, we could not monitor the CD spectra of the protein in the presence of the complexes below 220 nm, as they show strong absorption below this wavelength range and also due to observing high voltage values for the instrumental limitation within the mentioned wavelength range.^44^ None of the complexes cause any alteration in the secondary structure of BSA enabling the fact that though both the complexes interact with the protein, they have not induced any structural changes of the native state of BSA. To predict the probable binding site of the complexes within BSA, we have performed AutoDock based blind molecular docking study. The complexes locate them within BSA (Fig. S11a and b†). Moreover, the binding locale of both the complexes is in near vicinity with tryptophan and some hydrophobic amino acid residues of the protein (Fig. 8a and b). The observed binding energy from docking study also dictates that complex 1 has more affinity than complex 2, which well corroborate with our steady-state observations (Table S3†). The binding energies for the complex 1 and complex 2 with BSA were estimated as −24.27 kJ mol^−1^ and −23.65 kJ mol^−1^ respectively.

### DNA binding results

The changes in absorption maximum value of 5 μM of CT-DNA was governed by the addition of both the complexes and the results are shown in Fig. S16a & b.† It is observed that the intensity of the band for DNA slightly increases with addition of high concentration of the complexes. These clearly indicate that there is a little binding affinity of the complexes with the base pairs of CT-DNA.

**Fig. 9:**
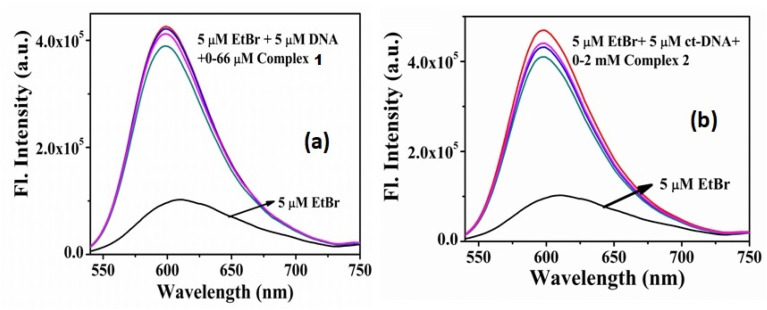
Addition effects of complexes 1 and 2 on the emission intensity of the CT-DNA bound EtBr at different concentrations.

In the fluorescence studies, CT-DNA bounded ethidium bromide (EtBr) shows an emission at 612 nm on excitation at 500 nm. On addition of the complex, almost no quenching of the EtBr-(CT-DNA) fluorescence intensity obtained for both the complexes (Fig. 9). This is further supported from the molecular docking studies of complex 1 and 2 with ct-DNA (Fig. S17 & S18†). The binding energies (change of free energy) for complex 1, 2 and EtBr with ct-DNA were found as −21.84, −21.21 and −36.21 kJ mol^−1^ respectively which reflects the weak capability of the complexes for quenching the fluorescence intensity of EtBr-(CT-DNA) adduct by replacing EtBr from this adduct. All these DNA binding studies clearly demonstrate no significant binding capability of the complexes with DNA.

### *In vitro* DPPH assay for antioxidant activities

**Fig. 10:**
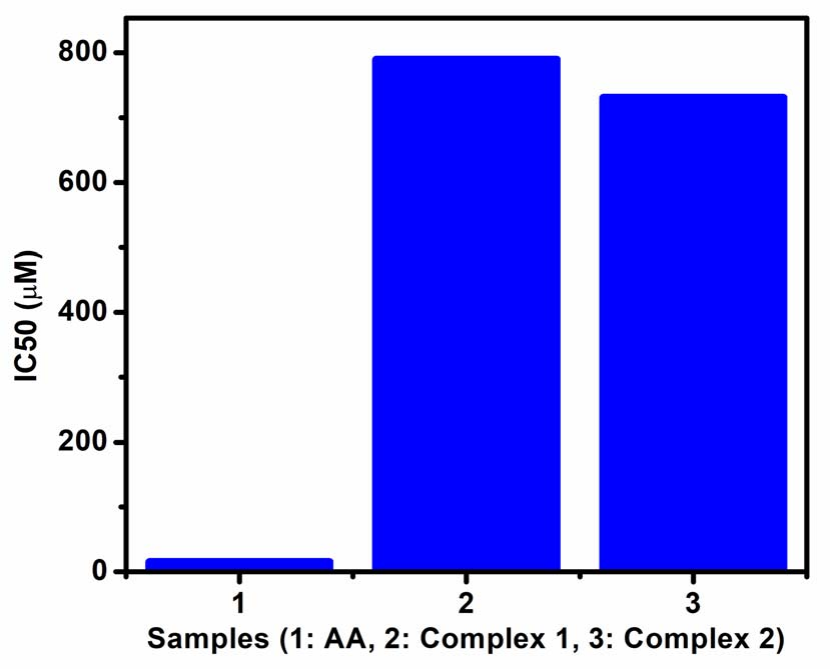
Comparison plot of IC50 values for ascorbic acid (AA), complex 1 and complex 2.

The oxidative assay of DPPH is used extensively for assessing the capability of radical scavenging activity or hydrogen donor's ability of complexes in terms of IC50 values (50% inhibition).^42^ The examined changes in the free radical scavenging ability of the test complexes on the basis of percentage inhibition are presented in Fig. S19a–c.† From the comparison plot of IC50 values (Fig. 10), it is shown that the studied complexes showed weak antioxidant activities with respect to ascorbic acid (AA) used as standard and it is also revealed that complex 2 showed slightly better antioxidant capacity than complex 1. The pictures which show color changes on gradual addition of AA and complexes to pure DPPH violet solution (Fig. S13b, S14b & S15b†) also support the above facts.

### *In vitro* cytotoxicity studies

**Fig. 11:**
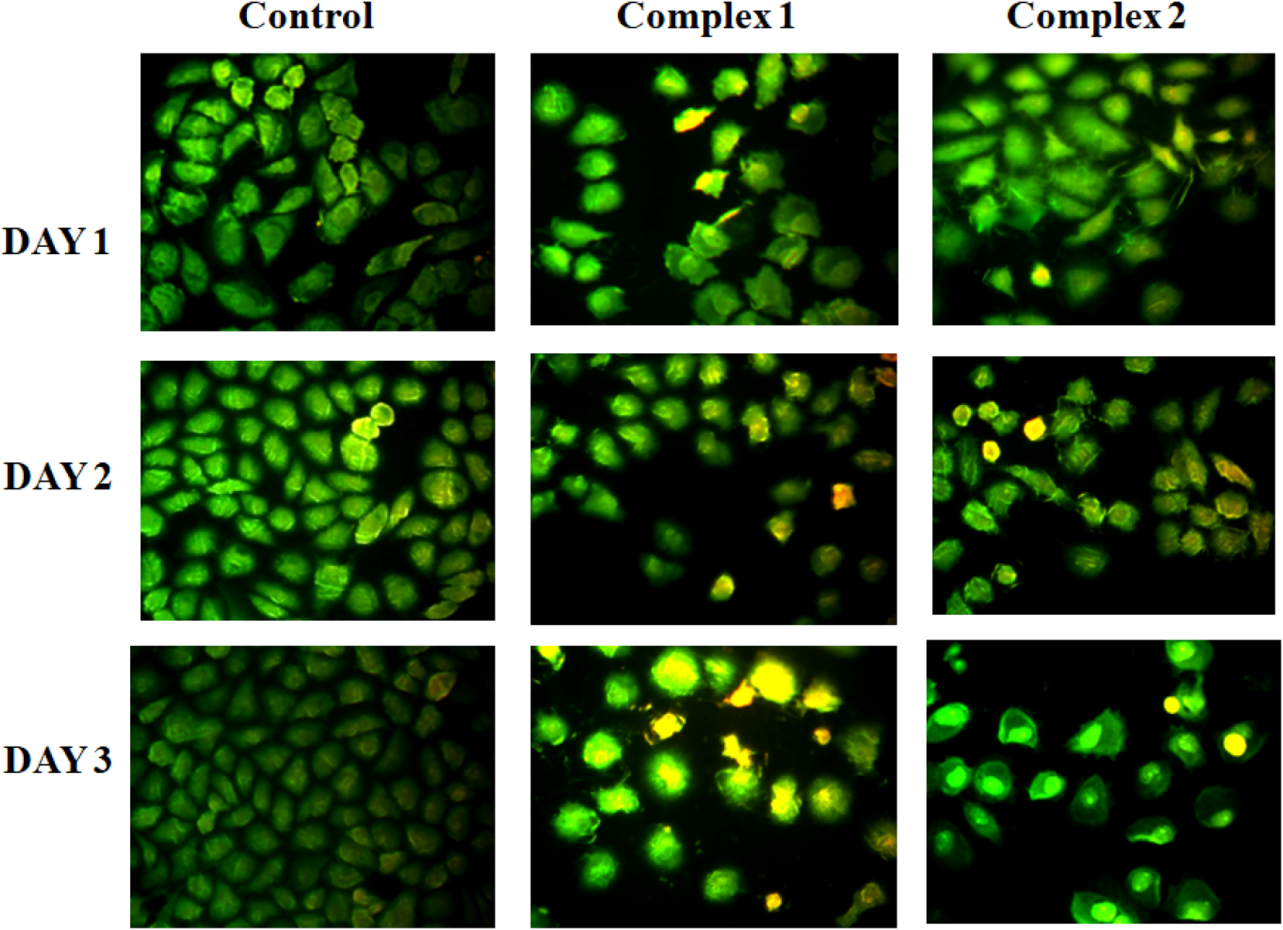
AO/EtBr staining fluorescent images for cancer cells.

Cytotoxic effect of the studied complexes against SiHa cancer cells and cell proliferation inhibition have been inspected *in vitro* after incubation of cells by fixed concentration of the complexes for 72 h using MTT assay. As control, the refined cells without sample are taken and 50 μg mL^−1^ of each complex was utilized for this study. Fig. S12† shows the results with mean ±1–1.5 standard deviation values with respect to individual experiments. Studies showed that for both the cases, presence of complex caused a momentous gradual decreasing of cell viabilities with time in cancerous cell lines than the control revealing their cytotoxic nature. To support these data additionally, fluorescent images were collected using AO and EtBr dye. AO and EtBr recognize the normal cells from apoptotic cells on the basis of cell membrane permeability. It is generally observed that AO shows green fluorescence and EtBr demonstrates red fluorescence on binding with DNA. Generally, dead cells shine red and permeable to both the AO and EtBr dye. Whereas, the living cells shine green and permeate only AO. After treatment, the comparative number density of cells can be visualized from fluorescent images of specimen staining with AO/EtBr (Fig. 11). Moreover, the colour of the cell after treatment gradually changes from green to yellow indicating cell apoptosis from day 1 to day 3. The cell viability obtained for the complexes after 72 h against the cancer cells is very much analogous to the reported values in literature.^48,49^ So, the complexes are significantly cytotoxic in nature and have potential to act as anticancer agent.

We have also checked the cytotoxicity of the studied complexes against normal cell besides cancer cell lines to ensure whether our complexes are toxic or not against normal cell. We have observed that the cell viability percentages of our complexes against 3T3-L1 cells (several normal cell lines of mouse) are higher (Fig. S20†) than cancer cell lines indicating that our complexes are less toxic with the normal cell but more toxic against cancer cell thereby demonstrating their uses as anticancer therapeutics.

## Conclusions

The present investigation derives from the syntheses and characterizations of Cu(ii) and Ni(ii) Schiff base complexes with respect to crystal structure, Hirshfeld surface, BSA interaction and cytotoxicity studies against SiHa cancer cell. Both the complex 1 and complex 2 show a distorted octahedral geometry in the crystal structure. From the experimental studies of BSA interaction it is observed that complex 1 shows greater binding competence with BSA than complex 2 whereas DNA binding results reveal the similar trend in DNA binding affinities for both the complexes with a small extent of binding capabilities. The DPPH assay studies demonstrate that the studied complexes possess weak antioxidant activities with respect to standard ascorbic acid (AA). Interestingly, these newly designed complexes depicted the *in vitro* anti-proliferative activities against SiHa cancerous cells. The strong protein binding affinity of the complexes apart from their weak DNA binding affinities may be responsible for the cytotoxicity against SiHa cancerous cell. Moreover, these complexes are less toxic for 3T3-L1 normal cell lines demonstrating their scope in chemo-therapeutics as specific anticancer components.

## Conflicts of interest

It is hereby confirmed that no conflicts of interest to be declared.

## Supplementary Material

RA-013-D2RA08341H-s001

RA-013-D2RA08341H-s002
