# Supplementary material for: Combined theoretical and experimental insights on DNA and BSA binding interactions of Cu(ii) and Ni(ii) complexes along with the DPPH method of antioxidant assay and cytotoxicity studies
Source: RSC Adv. 2023 Mar 8;13(11):7632–44. doi: 10.1039/d2ra08341h (PMC9993069; doi:10.1039/d2ra08341h)
Supplement: RA-013-D2RA08341H-s001 [file RA-013-D2RA08341H-s001.pdf]

Electronic Supplementary Material (ESI) for RSC Advances.
This journal is © The Royal Society of Chemistry 2023

Supporting Information

A combined theoretical and experimental insights on DNA and BSA binding interactions of Cu(II) and Ni(II) complexes along with DPPH method of antioxidant assay and cytotoxicity studies

Prasun Acharya[§], Arun Kuila[§], Ushasi Pramanik[¶], Venkatesha R. Hathwar[‡], Paula Brandao[¥], Saptarshi Mukherjee[¶], Swapan Maity[≠], Tithi Maity[†], Ribhu Maity[§], Bidhan Chandra Samanta[§]*

[§]*Department of Chemistry, Mugberia Gangadhar Mahavidyalaya, Bhupatinagar, Purba Medinipur-721425, West Bengal, India*

[¶]*Department of Chemistry, IISER Bhopal, Bhopal Bypass Road, Bhauri, Bhopal 462 066, Madhya Pradesh, India*

[‡]*School of Physical and Applied Sciences, Goa University, Taleigao Plateau, Goa 403 206, India*

[¥] *Departamento de Química /CICECO, Universidade de Aveiro, 3810-193 Aveiro, Portugal*

[≠]*School of Materials Science and Technology (SMST), Indian Institute of Technology (IIT), BHU, India*

[†]*Department of Chemistry, Prabhat Kumar College, Contai, Purba Medinipur-721401, West Bengal, India*

Corresponding address: *Department of Chemistry, Mugberia Gangadhar Mahavidyalaya, Bhupatinagar, Purba Medinipur-721425, West Bengal, India, Phone: 91-3220-270236, Email: bsmgm1977@gmail.com; bidhansamanta@yahoo.in (BCS)*

Statements of financial assistance: "Funding" (Order No. HRD-11011/161/2020-HRD-DBT, Dated 24/08/2020)

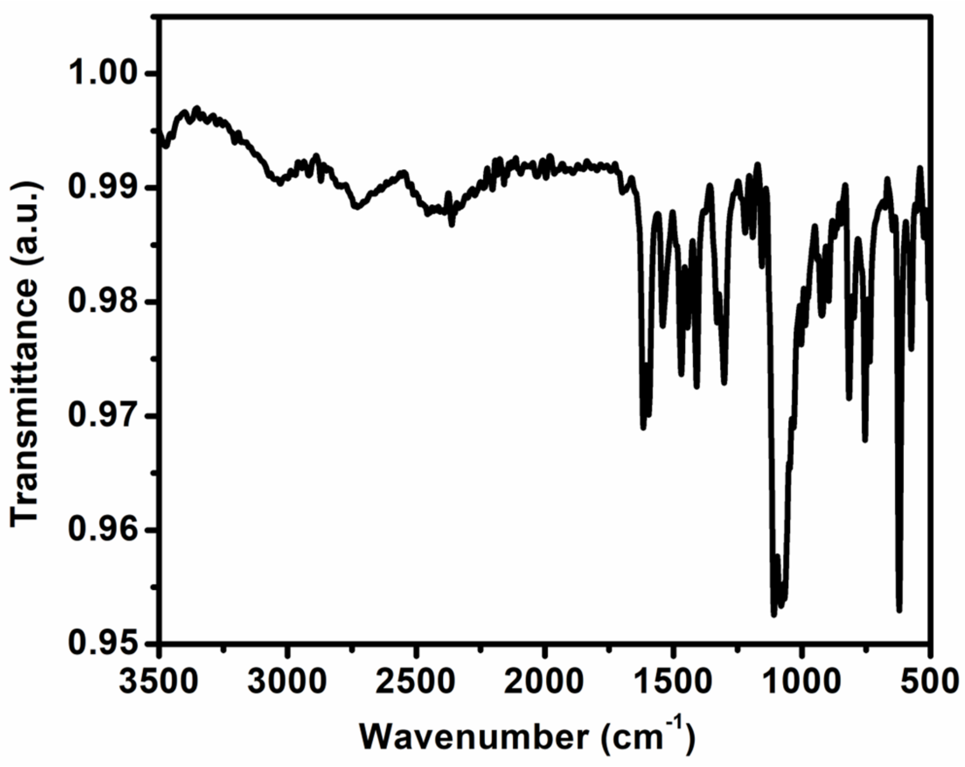

Figure S1 FTIR spectrum of complex 1

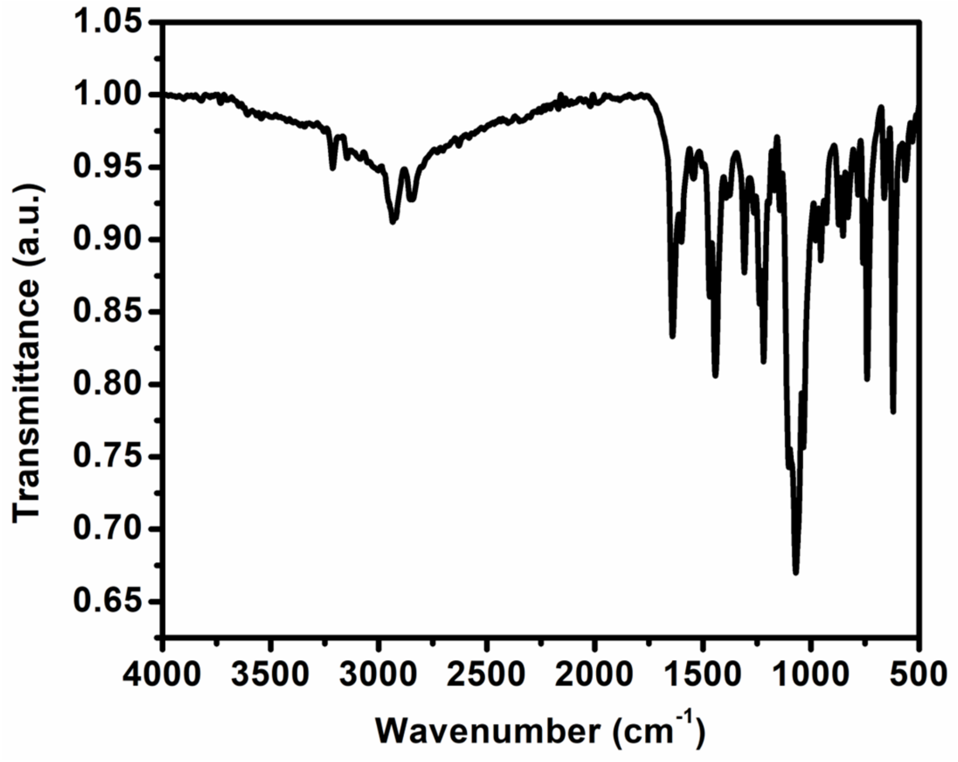

Figure S2 FTIR spectrum of complex 2

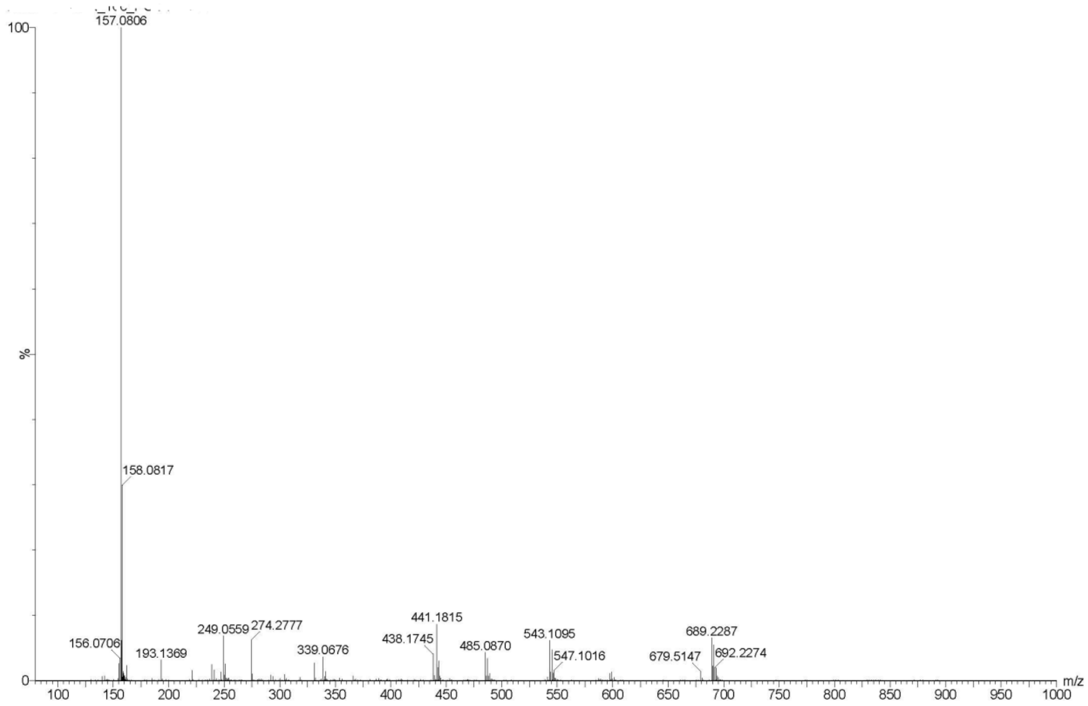

Figure S3 ESI-MS spectrum of complex 1

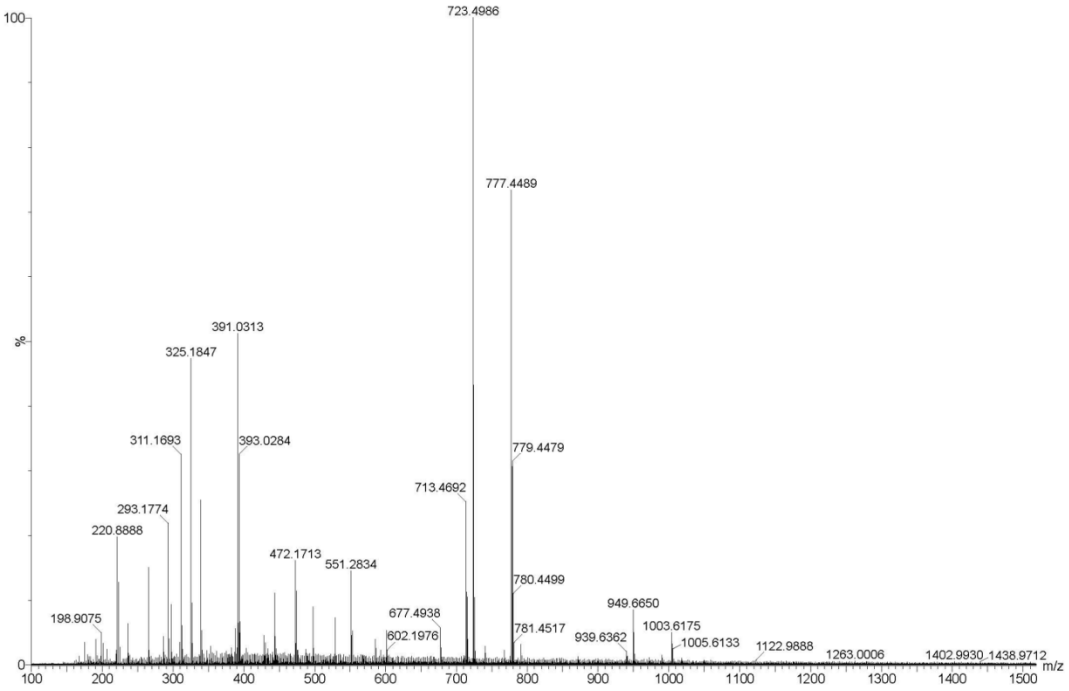

Figure S4 ESI-MS spectrum of complex 2

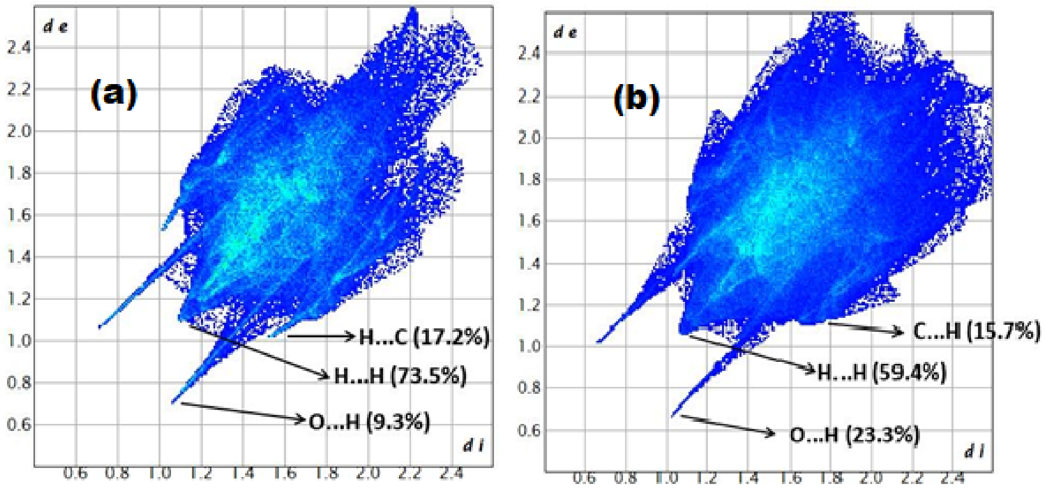

Figure S5 (a) The fingerprint plots with the percentage of contributions to the crystal structure for complex 1 and (b) for complex 2

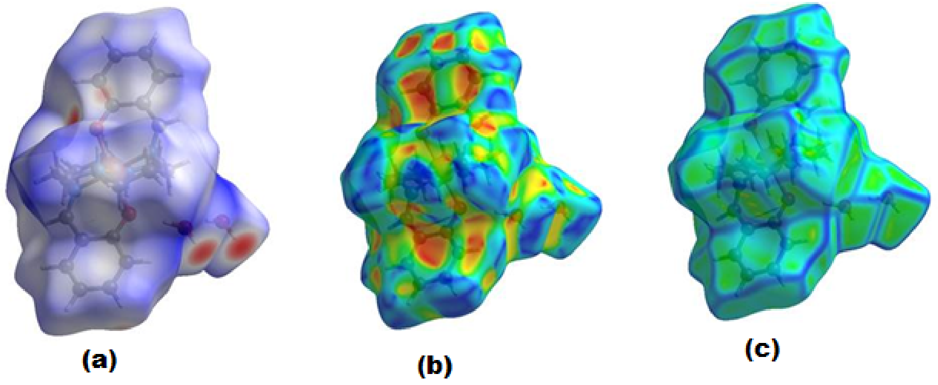

Figure S6 The Hirshfeld surfaces obtained by mapping (a) d_{norm} (b) shape-index and (c) curvedness properties for complex 1

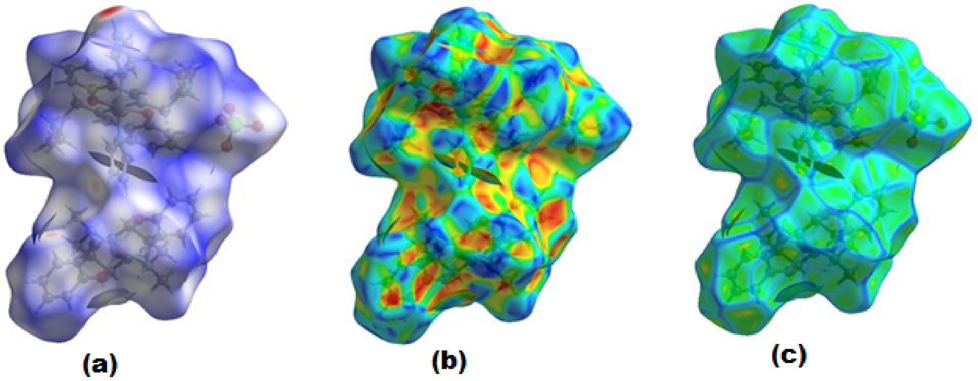

Figure S7 The Hirshfeld surfaces obtained by mapping (a) d_{norm} (b) shape-index and (c) curvedness properties for complex 2

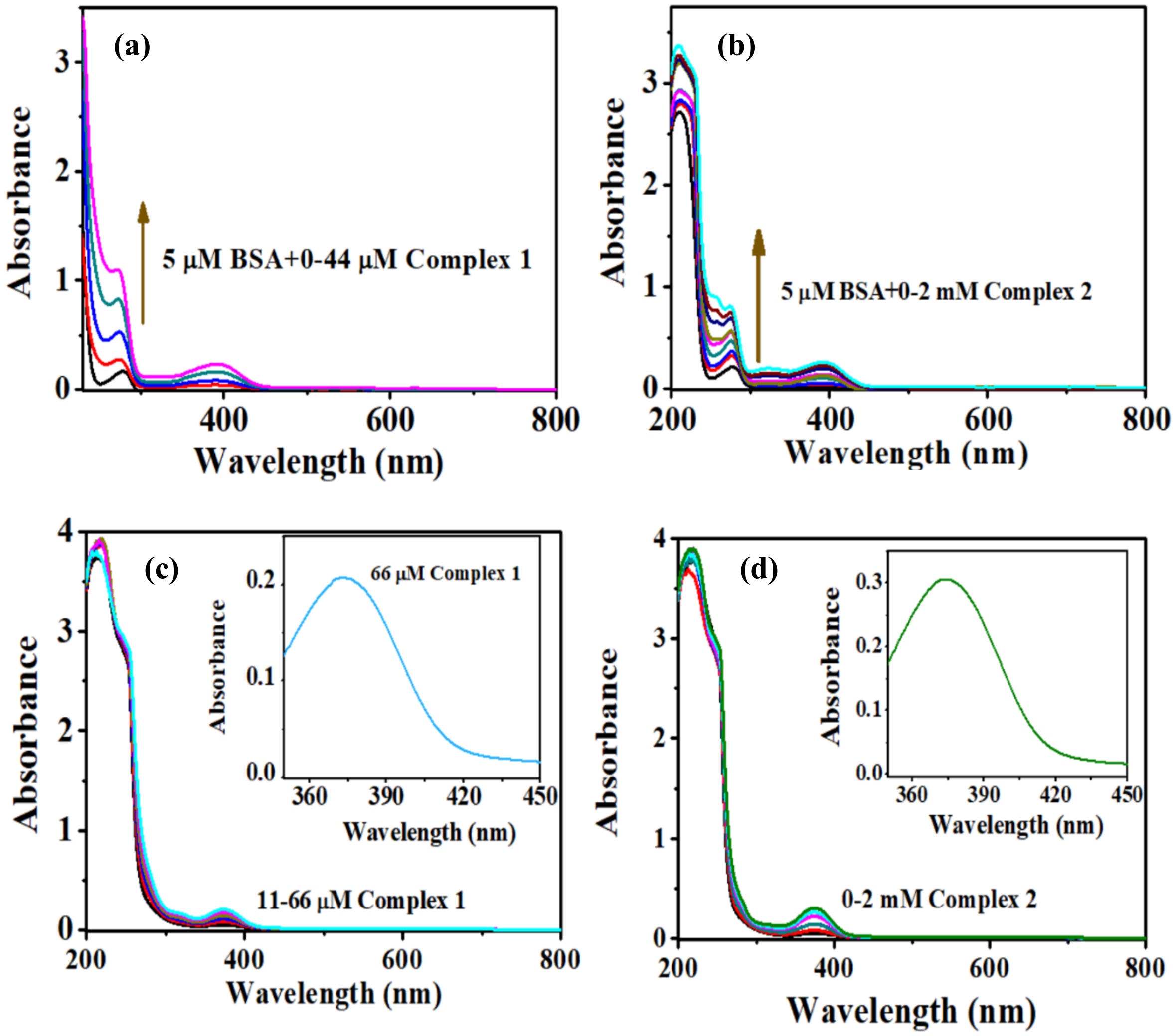

Figure S8 UV-Vis absorption spectrum of 5 μM BSA with increasing concentration of (a) complex 1and (b) complex 2.UV-Vis absorption spectrum of only (c) complex 1 and (d) complex 2

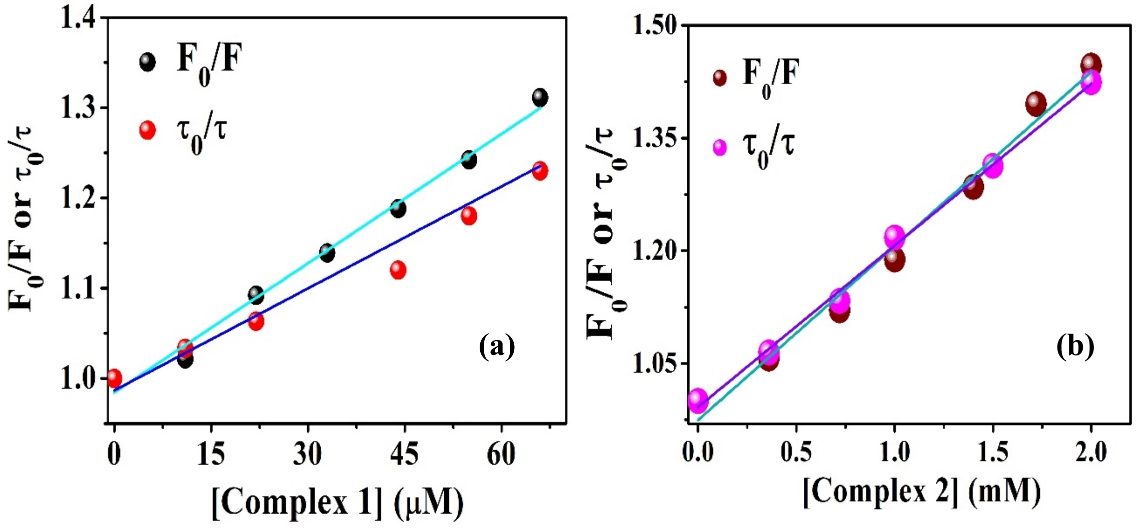

Figure S9 A plot of F0/F or τ0/τ as a function of concentrations of (a) complex 1 and (b) complex 2

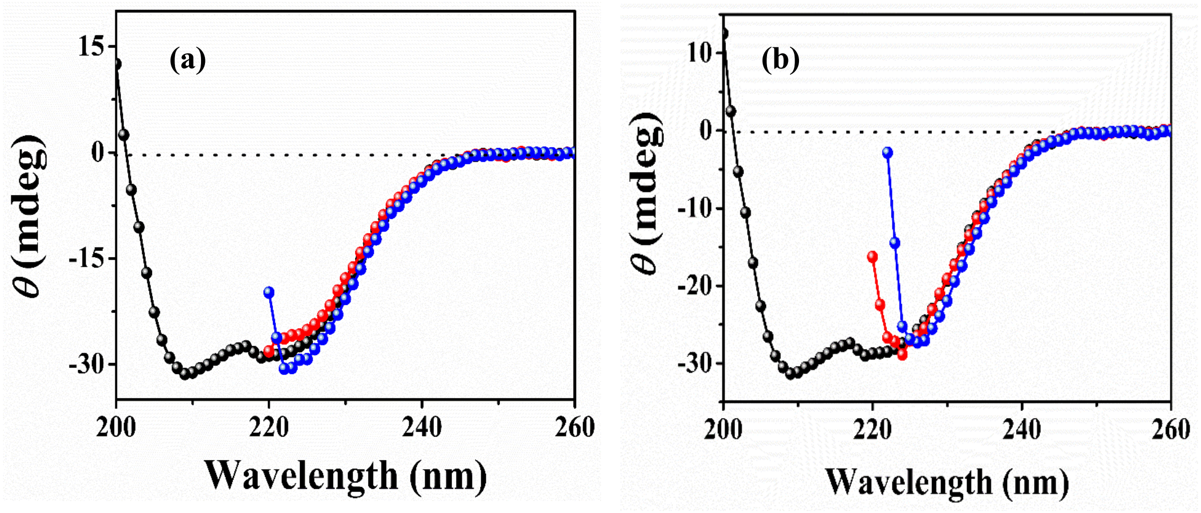

Figure S10 (a) CD spectra of 2 μM BSA (black) with increasing concentrations of complex 1 [13 μM (red) and 26 μM (blue) complex 1] as marked in the figure. (b) CD spectra of 2 μM BSA with increasing concentrations (432 μM; red and 864 μM; blue) of complex 2 as marked in the figure.

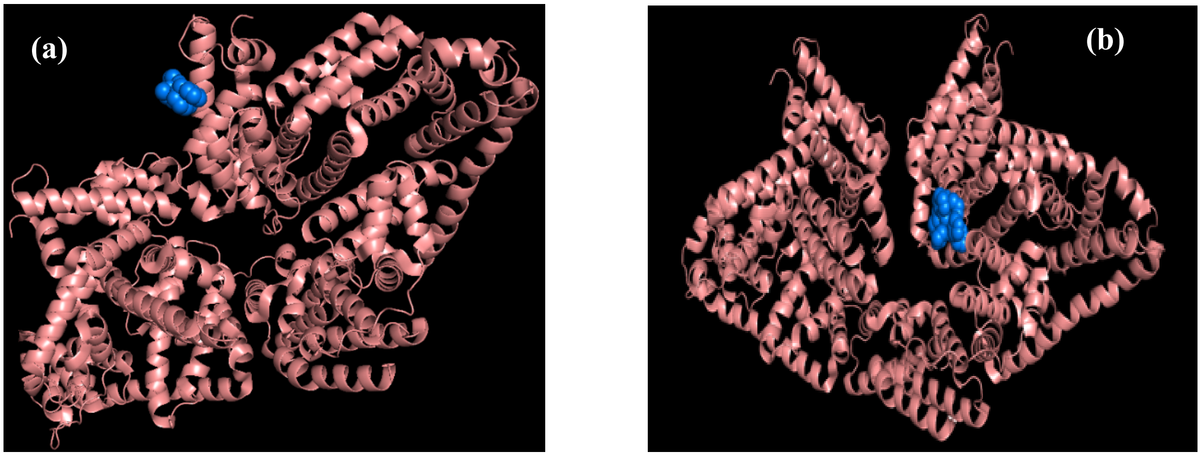

Figure S11 The best docked binding position of the (a) complex 1 and (b) complex 2 (shown as blue spheres) within BSA (orange ribbons)

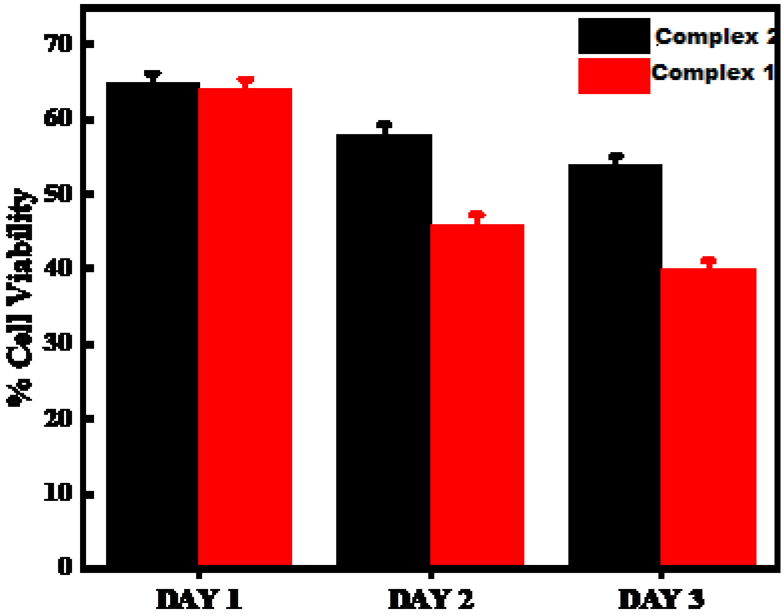

Figure S12 Cell viability percentage of the complexes against SiHa cells

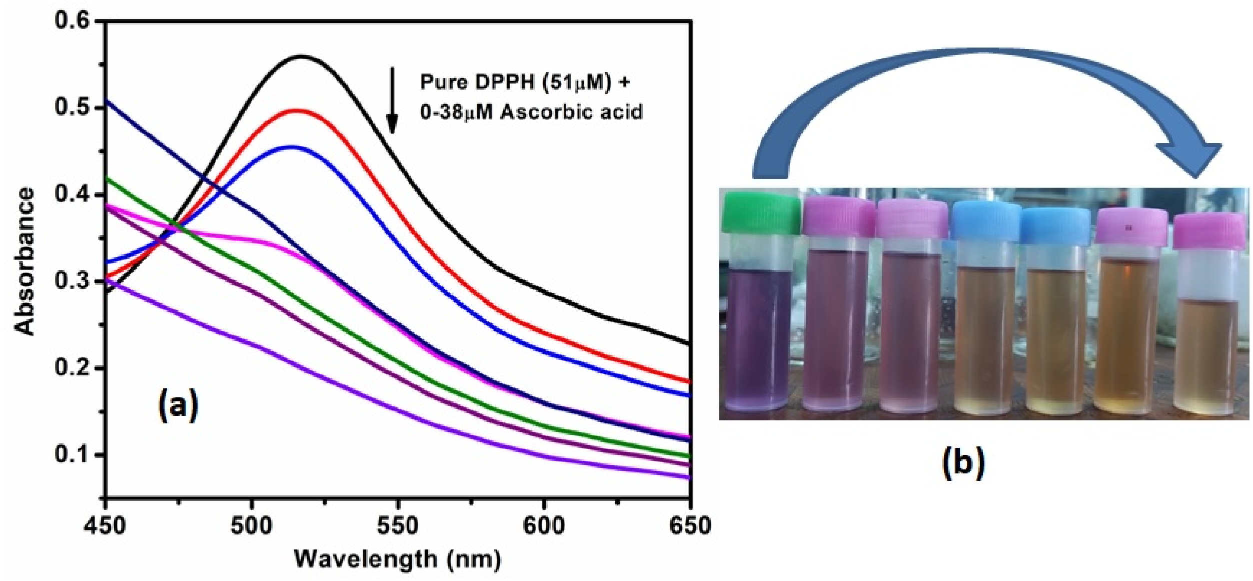

Figure S13 (a) Decrease in absorbance of DPPH with addition of ascorbic acid (b) Colour changes of pure DPPH solution with gradual addition of ascorbic acid

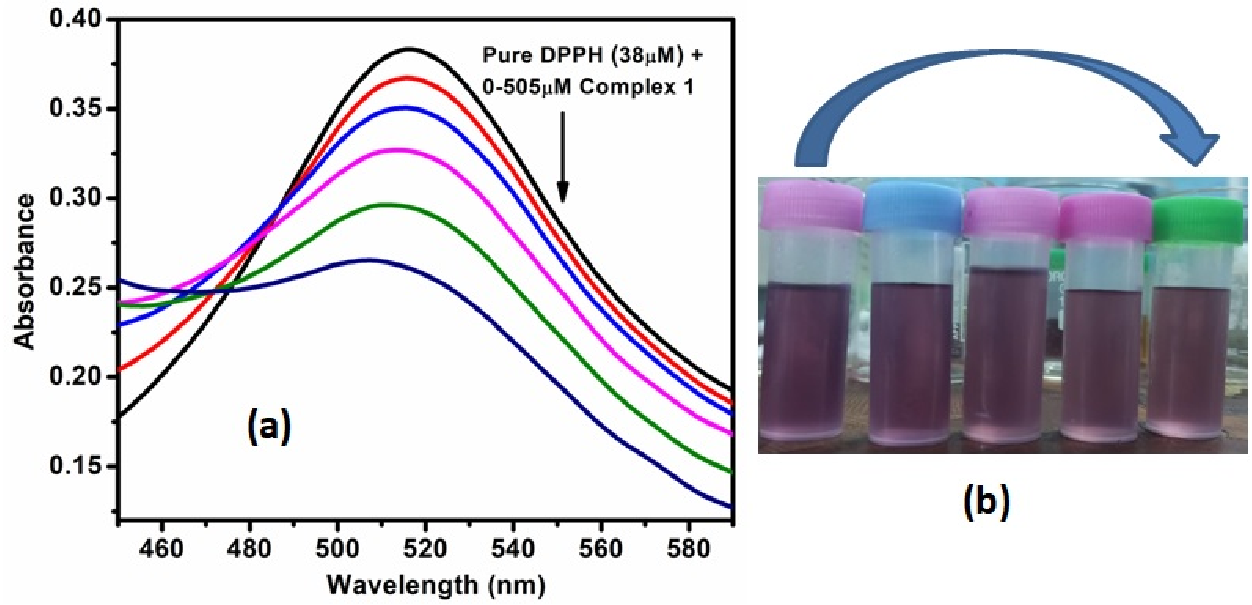

Figure S14 (a) Decrease in absorbance of DPPH with addition of complex 1 (b) Colour changes of pure DPPH solution with gradual addition of complex 1

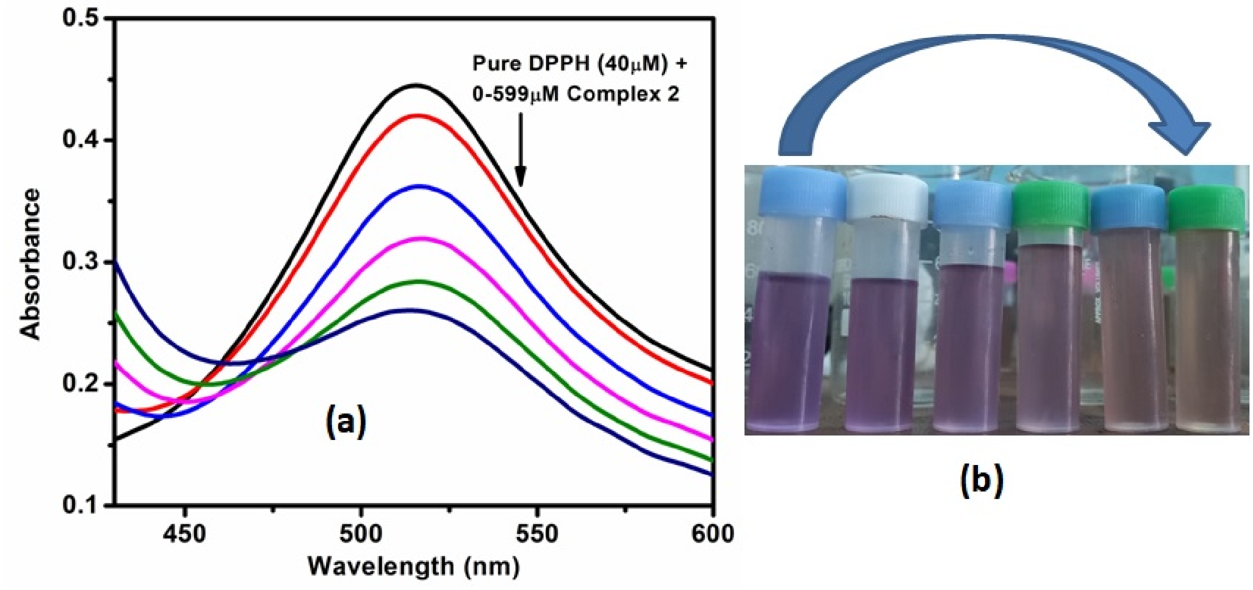

Figure S15 (a) Decrease in absorbance of DPPH with addition of complex 2 (b) Colour changes of pure DPPH solution with gradual addition of complex 2

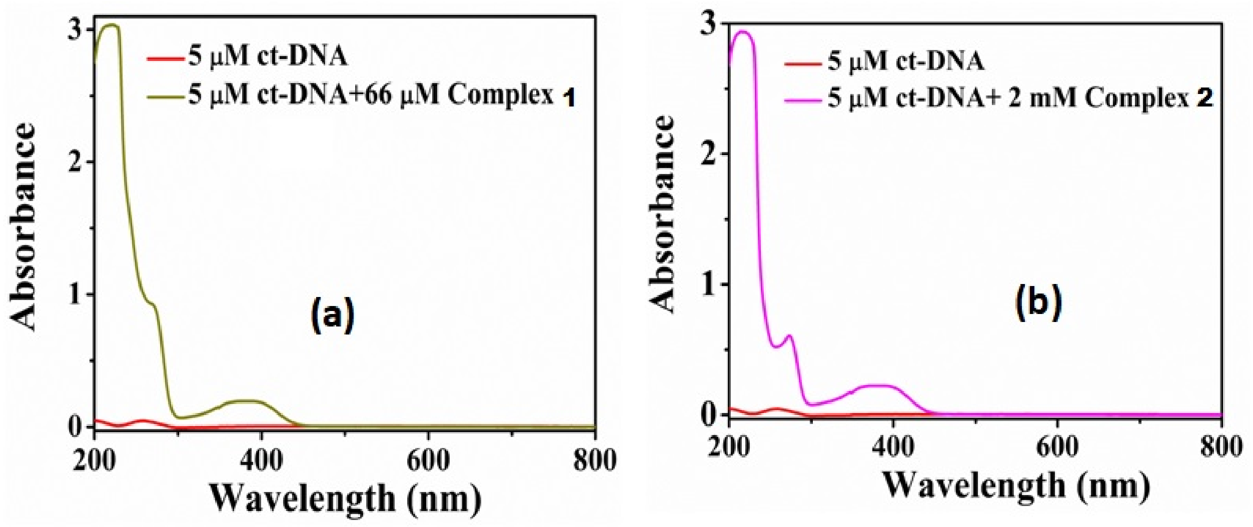

Figure S16 UV–Vis absorption spectra of ct-DNA (5 μM) upon the addition of (a) 66 μM for complex 1 and (b) 2 mM for complex 2

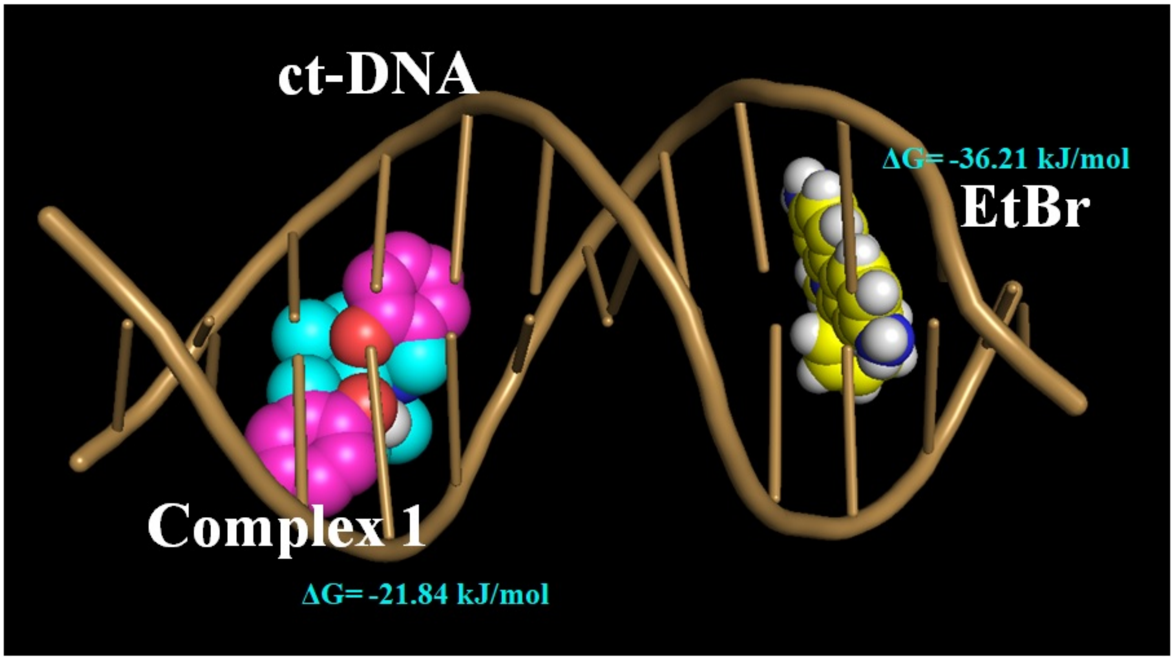

Figure S17 Docking pose of complex 1and EtBr with ct-DNA

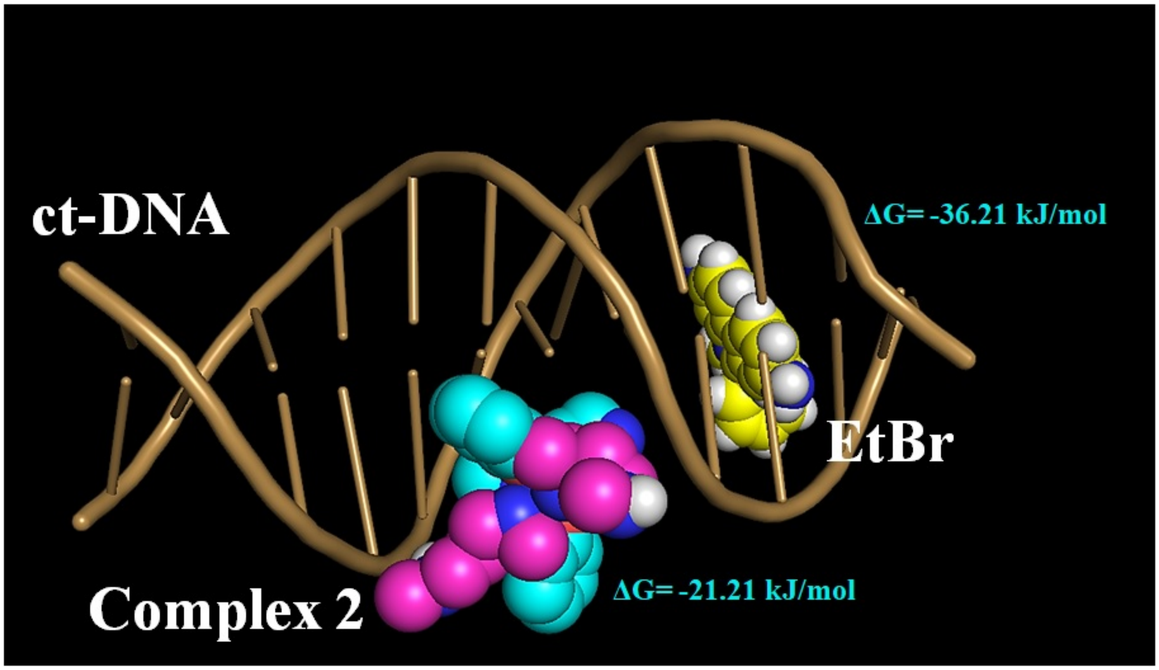

Figure S18 Docking pose of complex 2 and EtBr with ct-DNA

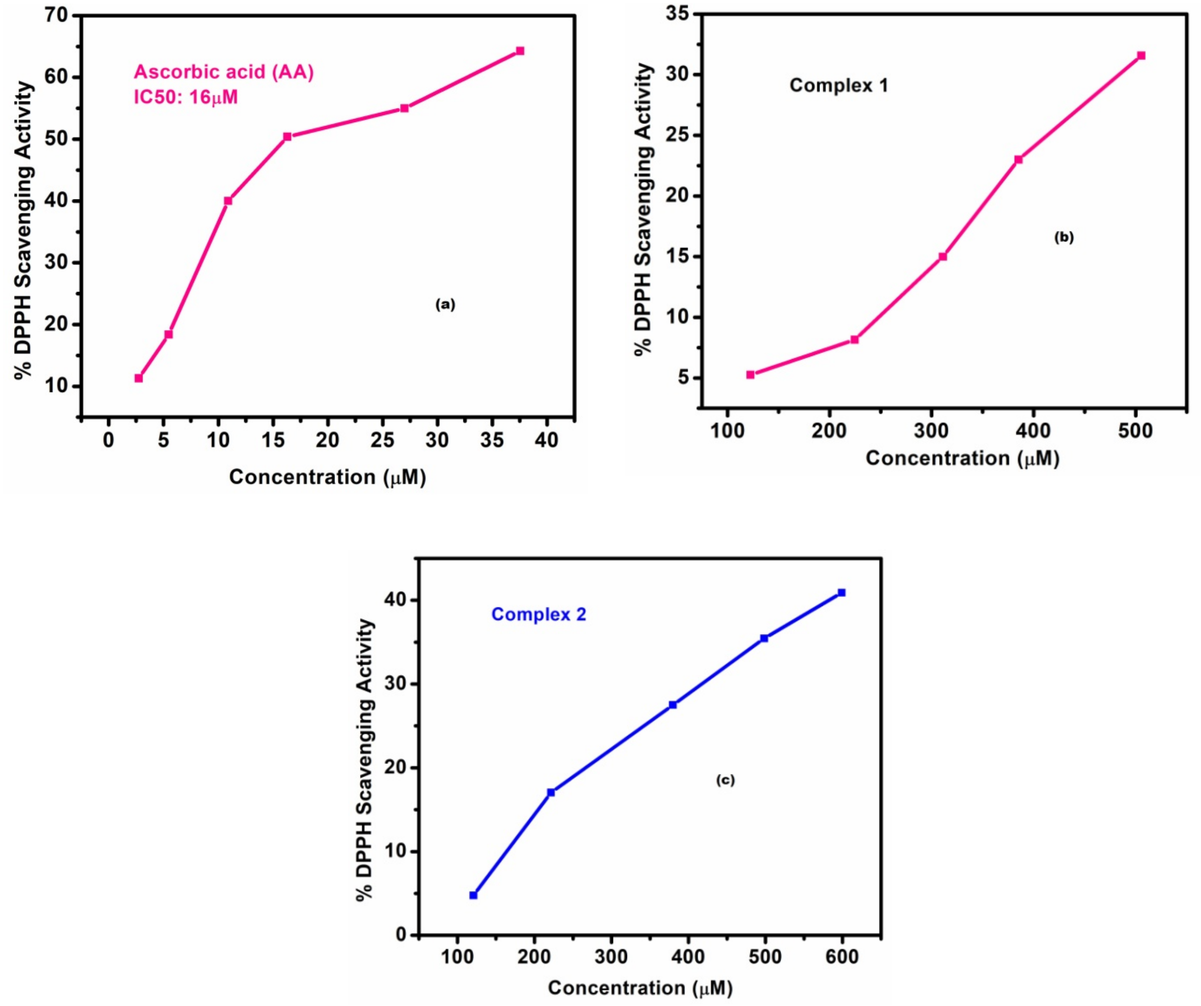

Figure S19 Antioxidant activity of (a) ascorbic acid (b) complex 1 (c) complex 2 by DPPH assay method

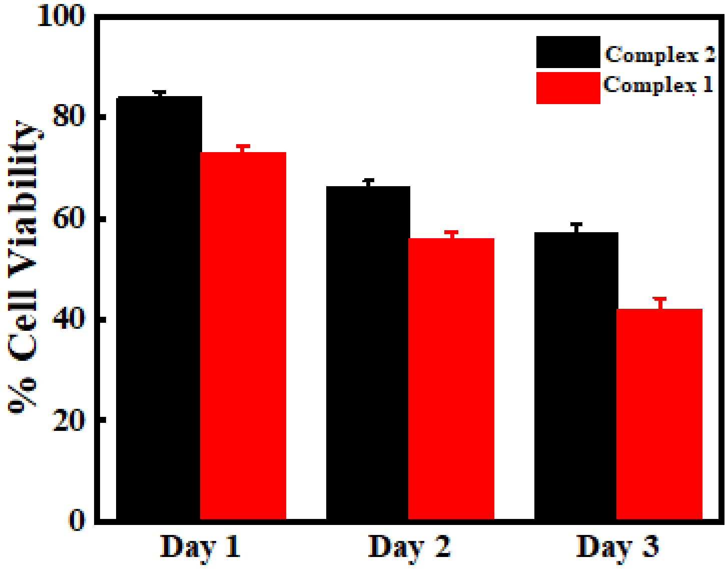

Figure S20 Cell viability percentage of the complexes against 3T3-L1 cells

Table S1 Selected bond lengths and bond angles for complex 1

Bond lengths		Bond Angles	
Cu1-O1	2.046(2)	O1-Cu1-N1	87.03(8)
Cu1-N1	2.060(2)	O1-Cu1-N2	87.13(8)
Cu1-N2	2.184(2)	N1-Cu1-N2	82.79(8)
Ni2-N5	2.074(2)	N3-Ni1-N5	86.59(6)

Table S2 Selected bond lengths and bond angles for complex 2

Bond lengths		Bond Angles	
Ni1-O1	2.059(1)	O1-Ni1-N1	86.46(5)
Ni1-N1	2.046(2)	O1-Ni1-N2	86.22(5)
Ni1-N2	2.181(2)	N1-Ni1-N2	83.23(6)
Ni2-O2	2.073(1)	O2-Ni1-N3	87.33(5)
Ni2-N3	2.103(1)	O2-Ni1-N5	89.05(6)
Ni2-N5	2.074(2)	N3-Ni1-N5	86.59(6)

Table S3 Binding parameters of the interaction between BSA and complexes

**Sample**	K_{SV} **(M^{-1})**	K_b **(M^{-1})**	***n***
Complex 1	(1.02±0.04) x 10^6	(2.11±0.21) x 10^4	0.91±0.04
Complex 2	(2.24±0.11) x 10^6	(1.05±0.05) x 10^3	0.88±0.01

Table 4 Lifetime parameters of tryptophan of BSA-complex 1 interaction

[Complex 1] (μM)	α_1	τ_1 (ns)	α_2	τ_2(ns)	$\langle\tau\rangle$ (ns)
0	0.13	3.10±0.10	0.87	6.67±0.12	6.21±0.08
22	0.14	2.93±0.04	0.86	6.38±0.07	5.90±0.02
44	0.16	2.74±0.08	0.84	6.20±0.05	5.64±0.09
66	0.18	2.23±0.05	0.82	5.80±0.05	5.15±0.05

Table S5 Lifetime parameters of tryptophan of BSA-complex 1 interaction

[Complex 2]	α_1	τ_1	α_2	τ_2	$<\tau>$ (ns)
0 mM	0.13	3.10±0.10	0.87	6.67±0.12	6.21±0.08
0.72 mM	0.13	2.87±0.06	0.85	6.27±0.10	5.70±0.05
1 mM	0.17	1.80±0.08	0.83	5.90±0.07	5.20±0.04
2 mM	0.20	1.60±0.04	0.80	5.85±0.10	5.00±0.04
